# How Do the Different Types of Carrier and Drying Techniques Affect the Changes in Physico-Chemical Properties of Powders from Chokeberry Pomace Extracts?

**DOI:** 10.3390/foods10081864

**Published:** 2021-08-12

**Authors:** Anna Michalska-Ciechanowska, Aleksandra Hendrysiak, Jessica Brzezowska, Aneta Wojdyło, Agnieszka Gajewicz-Skretna

**Affiliations:** 1 Department of Fruit, Vegetable and Plant Nutraceutical Technology, the Faculty of Biotechnology and Food Science, Wrocław University of Environmental and Life Sciences, Chełmońskiego 37, 51-630 Wrocław, Poland; 109211@student.upwr.edu.pl (A.H.); jessica.brzezowska@upwr.edu.pl (J.B.); aneta.wojdylo@upwr.edu.pl (A.W.); 2 Laboratory of Environmental Chemometrics, Faculty of Chemistry, University of Gdansk, Wita Stwosza 63, 80-308 Gdansk, Poland; agnieszka.gajewicz@ug.edu.pl

**Keywords:** *Aronia melanocarpa* L., by-products, sustainability, inulin, trehalose, polyphenols, HMF, unsupervised chemometric analysis

## Abstract

Chokeberry fruit, one of the richest plant sources of bioactives, is processed into different foodstuffs, mainly juice, which generates a considerable amount of by-products. To follow the latest trends in the food industry considering waste management, the study aimed to produce chokeberry pomace extract powders and conduct experimental and chemometric assessment of the effect of different carriers and drying techniques on the physico-chemical properties of such products. The PCA analysis showed that the examined powders were classified into two groups: freeze-dried (variation in case of moisture content, water activity, colour, and browning index) and vacuum-dried (bulk density). No clear pattern was observed for the physical properties of carrier added products. The sum of polyphenolics (phenolic acids, anthocyanins and flavonols) ranged from 3.3–22.7 g/100 g dry matter. Drying techniques had a stronger effect on the polyphenols profile than the type of carrier. Hydroxymethyl-*L*-furfural formation was enhanced by inulin addition during high-temperature treatment. Overall, the addition of maltodextrin and trehalose mixture for freeze drying and vacuum drying at 90 °C caused the highest retention of polyphenolics and the lowest formation of hydroxymethyl-*L*-furfural; however, an individual and comprehensive approach is required when the obtainment of high-quality chokeberry powders is expected.

## 1. Introduction

As of late, a new trend has become increasingly evident in the food industry, with consumers shifting their preferences from animal to plant-based products. This is mainly driven by consumers’ growing awareness of a healthy lifestyle, of which a balanced diet is an indispensable part, but also by ecological and ethical issues [1]. For this reason, the production and processing of plant products, mainly fruit and vegetables, has been increasing significantly for some time. However, this leads to the generation of an enormous amount of by-products, including pomace, the management of which is currently one of the biggest challenges for the food industry sector [2]. As it was reported earlier in the literature, fruit and vegetable pomace is a valuable source of numerous bioactive compounds [3,4]. One of the most frequently processed raw materials is black chokeberry (*Aronia melanocarpa* L.), which, due to its characteristic astringent taste, is not usually consumed as a fresh fruit. The main direction of its use is the production of juices, jams, or fruit wines, which results in significant amounts of by-products [5]. Due to its unfavourable sensory properties, it often remains wasted. However, a body of evidence has demonstrated that chokeberry pomace is a rich source of polyphenolic compounds, which have strong antioxidant properties, but has also indicated the beneficial effects in, among others, obesity, glucose metabolic disorders, pro-inflammatory conditions, hypertension, dyslipidaemia, etc. [6]. It has to be stressed that chokeberry pomace has almost an eight times higher content of polyphenols than juice [7]. Due to the proven health-promoting properties of chokeberry pomace, it can be an excellent raw material when developing functional foods [7]. In this setting, the processing of chokeberry by-products is of great importance and the production of a powdered form is new and one of the most promising alternatives for its utilisation, while at the same time being an effective tool for introducing sustainable food management [8,9]. One of the interesting approaches is to obtain powders from chokeberry pomace extracts. This type of product, due to its easy-to-handle form, possible high solubility (in contrast to pomace), and a relatively high microbial stability, is an attractive additive to other foodstuffs as a sustainable natural colouring or functional enrichment agent [5,10]. However, in order to retain the satisfactory amount of selected polyphenolics, a correctly chosen extraction method is essential, as are the subsequent steps: solvent evaporation, purification of the extracts on a polymer-bed-type Amberlite XAD, and next solvent evaporation leading to a purified pomace polyphenolic extract obtainment [11]. Moreover, in order to acquire the powdered form, it is necessary to carry out drying, which may significantly affect the physical properties, but also can lead to alterations of the chemical composition, particularly in the profile of polyphenolic compounds. For this reason, the choice of an appropriate drying technique and its parameters is pivotal to maintain a relatively high possible content of polyphenolic compounds in the final product [12]. Freeze drying, as a low-temperature water removal process, is considered to be the least intrusive with the slightest impact on the transformation of the dried matrix [13]. On the other hand, recent studies by Michalska-Ciechanowska et al. [12] on black chokeberry juice demonstrated that the application of high temperatures during drying can positively influence the polyphenolic profile, leading to the release of significant amounts of selected bioactive constituents from more complex structures [12]. One technique that enables the use of high drying temperatures while excluding the effects of oxygen on the dried matrix is vacuum drying [14]. However, the thermolability of polyphenols, especially anthocyanins, to thermal processes, as well as the different yields for obtaining powders depending on the raw material composition, induce the necessity of using a carrier additive in the drying process [15]. Substances of carbohydrate origin, such as maltodextrin, are widely used for this purpose and have proven protective properties, allowing for an increased retention of anthocyanins at high drying temperatures compared with other carriers [14]. Further interesting additives include trehalose, which has been found to be inactive in the Maillard reaction due to its non-reducing properties, and inulin, which is well known for its functional properties [16]. Importantly, the selection of carrier is of high importance as it was previously demonstrated that the type and concentration of selected carriers may not only cause a decrease in the polyphenolics content, but may also accelerate the formation of undesirable process contaminants, especially in fruit-based matrices [12,14]. Taking the above into consideration, it is hypothesised that the drying techniques and type of carrier will simultaneously moderate the polyphenolic composition in chokeberry pomace extract powders. Thus, this study aimed to evaluate different drying techniques and parameters as well as carrier types on the alteration in the polyphenolics composition, antioxidant capacity, and formation of Maillard reaction and caramelisation products in chokeberry pomace extract powders.

## 2. Materials and Methods

### 2.1. Materials

The material used in the study was composed of chokeberry fruits (approximately 70 kg) obtained from Rolniczo-Sadownicze Gospodarstwo Doświadczalne ‘Przybroda’ (Rokietnica near Poznań, Poland). The fruits were ground in a Thermomix (Wuppertal, Vorkwek, Germany) and pressed on a hydraulic press (SRSE, Warszawa, Poland). The pomace gained was frozen before the extraction process at −20 °C.

### 2.2. Methods

#### 2.2.1. Extraction Procedure

The extraction of polyphenolic compounds from chokeberry pomace (initial moisture content of 44.45 ± 0.01%) was performed according to the patent Oszmiański and Krzywicki [17]. Thawed pomace (approximately 10 kg) was mixed with 30% acetone (1:4, *w*/*v*) and sonicated for 15 min. The solution was left for 24 h and sonicated again for 15 min. The acetone was evaporated (Unipan 350P, Warsaw, Poland) and the solution gained in a quantity of approximately 17 L (4.9 ± 0.07 Bx) was introduced into the Amberlite XAD-16 (Brenntag, Poland) according to the procedure of Kammerer et al. [11] in order to recover the selected polyphenolics in the extracts [18]. This resulted in approximately 2.5 L of final solution (6.9 ± 0.1 Bx), which was submitted to the formulation of the drying compositions (control, samples with carrier addition). The procedure was performed in duplicate (*n* = 2).

#### 2.2.2. Preparation of Chokeberry Pomace Extract Powders

The pomace extracts were mixed with carriers, i.e., maltodextrin (M) (DE 9.3; PEPEES S.A, Poland), inulin (I) (Beneo-Orafti, Belgium), trehalose (T) (Hayashibara, Co., Okayama, Japan), and their mixtures maltodextrin-inulin (M-I), maltodextrin-trehalose (M-T), and inulin-trehalose (I-T) at the level of 10% (*w*/*w*) (chosen on the basis of experimental work). No carrier was added to the control sample. Prepared solutions were submitted to drying processes: freeze drying at −60 °C/+ 24 °C for 24 h (FreeZone freeze dryer, Labconco Corp., Kansas, MO, USA), and vacuum drying at 60 and 90 °C for, respectively, 22 and 16 h (Vacucell 111 Eco Line, MMM Medcenter Einrichtungen GmbH, Germany). The drying processes were performed in duplicate (*n* = 2). After the drying, the obtained powders were vacuum packed and stored at −20 °C until analysis.

#### 2.2.3. Physical Properties

##### Moisture Content

The moisture content (*Mc*) was determined in duplicate (*n* = 2) [12] at 80 °C. The results were expressed as %.

##### Water Activity

The water activity (*a_w_*) was done at 25 °C in duplicate (*n* = 2) using the water activity meter Dew Point Water Activity Meter 4TE (AQUA LAB, Pullman, WA, USA).

##### Bulk Density

The bulk density (ρ_b_) of powders was performed in duplicate (*n =* 2) using a graduated cylinder (10 cm^3^) and laboratory scale. This was calculated as follows:

(1) $$\rho_{b}=\frac{m}{{\;V}_{b}}$$

m—mass of the powder, V_b_—volume of the powder

##### Colour

The colour of the powders was measured in triplicate (*n* = 3) using a Minolta Chroma Meter CR-400 colorimeter (Minolta Co. Ltd., Osaka, Japan) according to the CIE L*a*b* system. Based on the results, the browning index (BI) was calculated according to the equation described by Mexis and Kontominas [19]:

(2) $$\mathrm{BI}=\frac{\left[100\left(x\;-0.31\right)\right]}{0.17\;}$$

where:

(3) $$x=\frac{a^{*}+1.75L^{*}}{5.64L^{*}+a^{*}-3.012b^{*}\;}$$

#### 2.2.4. Chemical Properties

##### Preparation of Extracts

The extraction of polyphenolics from the chokeberry pomace powders was performed according to the procedure described by Wojdyło et al. [20]. Samples for qualitative and quantitative determination of these compounds and hydroxymethyl-*L*-furfural (HMF) by Ultrahigh Performance Liquid Chromatography were done in duplicate (*n* = 2) and extracted with 1.7 mL of aqueous solution of MeOH (30%; *v*/*v*) with ascorbic acid (0.2%) and 0.1% CH_3_COOH while those for antioxidant capacity analyses (*n* = 2) were prepared with 1.7 mL of MeOH (80%; *v*/*v*) with HCl (1 mL/L). All samples were sonicated for 15 min and refrigerated at 4 °C for 24 h. After this time, the samples were ultrasonicated again (15 min) and then centrifuged (19,515× *g*, 20 °C; MPW-251, MPW Med. Instruments, Poland). The obtained extracts were subjected to further analyses.

##### Qualitative and Quantitative Determination of Polyphenolics and Hydroxymethyl-*L*-furfural

The qualitative and quantitative analyses of polyphenolics were performed using an Acquity UPLC system (Waters, Milford, MA, USA) with a PDA detector, equipped with a binary pump system and a solvent manager. The separation of the individual compounds was done at a flow rate of 0.42 mL/min in an ACQUITY BEH C_18_ analytical column (100 mm × 1.7 μm, Waters, Milford, MA, USA). The column was conditioned with acetonitrile (100%) and an aqueous solution of acetonitrile (10%; *v*/*v*). The separation was performed by the gradient elution method using 4.5% formic acid (solvent A) and acetonitrile (solvent B): 0–10 min linear gradient, 1–15% solvent B; 10–11.5 min linear gradient, 25–100% solvent B. Anthocyanins, flavonols, phenolic acids, and HMF were detected at λ = 520 nm, 360 nm, 320 nm, and 280 nm, respectively. The determination was performed in duplicate (*n* = 2). The polyphenolics were identified by LC-MS QTof and assessed using the MassLynx 4.0 ChromaLynx Application Manager software [21]. Results were expressed as g/100 g dry basis (db) for individual polyphenols and as μg/100 g of db for HMF.

##### Antioxidant Capacity

The antioxidant capacity of polyphenolic extracts from chokeberry pomace extract powders was evaluated by ABTS^+^ radical cation scavenging [22] and FRAP [23] by *in vitro* assays using a Synergy H1 spectrophotometer (BioTek Instruments Inc., Winooski, VT, USA). The determination was made in duplicate (*n* = 2) and the results were expressed as mmol Trolox equivalent (TE) per 100 g of db.

#### 2.2.5. Statistical Analysis and Chemometrics

The results were statistically analysed using STATISTICA 13 software (StatSoft, Tulsa, OK, USA). One-way analysis of variance (ANOVA) was performed (*p* < 0.05) and the Tukey post hoc test (also referred to as the Tukey’s honestly significant difference (Tukey HSD)) was applied to compare the pairwise differences between group means (*p* ≤ 0.05). To investigate the dependence between the selected variables, Pearson’s correlation coefficient was also calculated.

To identify the direction that maximises the variance of the projected data and to explore the trends and relations between observations and variables simultaneously, principal component analysis (PCA) [24] was performed using the ‘factoextra’ R package [25]. The radar plots were prepared using the ‘fmsb’ R package [26], allowing differences in sub-groups to be visualised.

## 3. Results and Discussion

### 3.1. Physical Properties

#### 3.1.1. Moisture Content

The moisture content (*Mc*) of the analysed powders ranged from 0.47% to 7.59% for the products gained, respectively, after vacuum drying at 90 °C and after freeze drying with the addition of trehalose (Table 1). In general, the average moisture content of the control samples (no addition of carriers) was lower by approximately 29% when compared to the average moisture content of the products gained with the addition of carriers. The drying techniques and parameters applied for the powders’ preparation had a significant effect on this parameter: the higher the drying temperature, the lower the *Mc* of the powders [27].

When the drying techniques and carrier type were concerned, it was noted that during the freeze drying process, the addition of maltodextrin resulted in the lowest *Mc,* whereas the addition of trehalose led to the highest moisture content of the powders (Table 1).

In the case of freeze and vacuum drying at 60 °C, the powders gained with the addition of inulin had a lower moisture content than the samples obtained with the addition of its mixes (M-I and I-T). However, when vacuum drying at 90 °C was considered, the powders obtained with the addition of inulin and its mixes had the highest *Mc* when compared to the rest of the applied carriers. It can be concluded that the type of carrier might influence the moisture content due to the different water-holding capacity, which could be additionally altered by the drying technique used for the powders’ preparation [28]. However, taking into account the moisture content of the carriers (i.e., *Mc* of M: 10.07 ± 0.93; *Mc* of I: 3.05 ± 0.01; *Mc* of T: 1.04 ± 0.04; *Mc* of M-I: 5.52 ± 0.01; *Mc* of M-T: 3.68 ± 0.18; *Mc* of I-T: 2.66 ± 0.13), it can be observed that *Mc* of the powders was influenced more by possible interactions between the compounds present in the extracts and the constituents of the carriers (Table 1). This was also confirmed by the lack of a significant correlation between the moisture content of the carriers themselves and *Mc* of the powders gained with their addition.

#### 3.1.2. Water Activity (*a_w_*)

In all the analysed powders, the water activity values were below 0.45 (Table 1), indicating that these products can be considered as stable from a biochemical and microbiological point of view [29]. In the case of the controls and powders with the addition of carriers and their mixtures, the water activity values were higher after the freeze drying process than in the products gained after vacuum drying at 60 and 90 °C [27]. This might be due to the more porous structure of the products gained after freeze drying, when compared to the other drying technique used [30]. Similarly to da Silva Calvaho et al. [29], the type of carrier influenced *a_w_* of the powders. In the products obtained with the application of maltodextrin and its mixes for freeze drying, I and I-T for vacuum drying at 60 °C and T for vacuum drying at 90 °C resulted in higher water activity in the products obtained. A positive correlation between water activity and moisture content was noted (*r* = 0.67) (Figure S1). A similar observation was made in case of apple juice powders [27]. In general, the application of different carriers and their mixture led to alterations in the water activity of the samples, which was also dependent on the drying technique used. There are reports suggesting that in relation to the type of carrier used for drying, the formation of a crust on the outer layer of samples was observed, which influences the water activity values in the products gained [31]. Thus, water activity may be connected with the physical changes that occur on the surface of the samples during drying that might differ in terms of their carrier properties.

#### 3.1.3. Bulk Density

The bulk density of chokeberry pomace extracts powders ranged from 0.09 g/cm^3^ to 0.83 g/cm^3^ for, respectively, the control sample gained after freeze drying and powders obtained after vacuum drying at 90 °C with the addition of I-T (Table 1). When the control samples and products gained with different carriers were concerned, the average value of bulk density was the lowest in the case of controls, whereas the highest bulk density was noticed for powders gained with the addition of carriers, especially when inulin and its mixtures were considered (M-I, I-T). In general, as observed by Michalska and Lech [27], freeze drying resulted in lower values of the bulk density of powders gained followed by VD at 60 °C and VD at 90 °C, except powders produced with trehalose. The addition of trehalose to pomace extract formulations caused the lowest bulk density of powders gained after VD at 60 °C and VD at 90 °C. This might be connected with the higher soluble solids content in the formulation containing trehalose [32] during the freeze drying process and/or with its different behaviour when a relatively high temperature of drying was applied. It could also be related to the fluidizing properties of this carrier [33] as trehalose, due to its chemical composition, might react differently with the compounds present in the extracts additionally moderated by the vacuum drying. What is more, the influence of the carrier type on the bulk density of powders was also noticed in the case of cranberry juice [34]. Taking the above into consideration, the selection of a carrier for powder preparation should also consider the carrier physico-chemical properties as it may moderate the physical properties of the material subjected to drying to a different extent. For the analysed powders, a negative correlation (*r* = −0.60) between bulk density and water activity (Figure S1) was noted, indicating that the specific structure of the powders gained by different drying techniques might differently influence the ability of samples to trap the water molecules [35].

#### 3.1.4. Colour

The colour parameters measured in terms of L*, a*, and b* values as well as the browning index (BI) are indicated in Table 1. In general, powders gained after the freeze drying process were the lightest, with the exception of samples prepared with trehalose, whereas vacuum drying at 60 and 90 °C resulted in slightly lower values of coordinate L*. In the latest samples, trehalose addition caused the highest values of coordinate L* in the analysed products. The type of carrier had a significant impact on the lightness of the powders gained [36], which was also moderated by the drying technique used. In general, when the values of coordinate a* were concerned, it was noted that the carrier addition altered the level of red pigments in chokeberry powders. Contrary to the juice powders [37], the 10% addition of carrier led to the obtainment of products that were visually similar to the control samples. Thus, the addition of carriers, besides an improvement of the efficiency of the sustainable powder production [38], may not visually alter the colour of the products gained. In this case, the composition of chokeberry pomace extracts played a key role in colour preservation as the addition of carriers into the chokeberry juice modified the lightness to a high extent [37].

The strongest retention of red pigment was noted after freeze drying when compared to samples gained after vacuum drying, except samples prepared with the addition of trehalose and its mixes (M-T, I-T). In general, the application of vacuum drying for the powder preparation caused a decrease in the coordinate a* values, indicating the influence of the high-temperature processing on the thermolability of the red components [39]. The strongest pigment degradation was noted in the case of powders produced with maltodextrin and its mixes (I-M; I-T). The addition of I and I-T seems to prevent deterioration of the coordinate a* values during vacuum drying. It was observed that the b* parameter was the highest in the control samples (no addition of carrier), regardless of the drying technique used for their preparation (Table 1). The addition of carriers led to a decrease in the coordinate b* values, which was the strongest in the case of trehalose application for the freeze drying process. This might be connected with the presence of natural colour compounds in the chokeberry pomace extracts (an average for FD: 3.37), as vacuum drying at 60 °C (an average: 0.97) and 90 °C (an average: 0.71) resulted in significantly lower values of parameter b*. In the current study, the browning index was applied for determination of overall alterations in the browning colour [40]. In contrast to sea buckthorn powders [41], the highest values of BI were noted for freeze-dried samples, regardless of the type of carrier used for their preparation. It was assumed that the dominant reddish colour could mask the brown pigments present in the analysed powders [42]. Additionally, the BI values might result from the complexes formed from polyphenols [43]. The application of vacuum drying for chokeberry pomace extract powder production caused a decrease in BI when compared to freeze drying. The addition of maltodextrin and its mixes (M-I, M-T) led to lower values of BI. Additionally, a strong correlation between BI and a* (*r* = 0.82) as well as b* (*r* = 0.83) confirmed the masking effect of the reddish and bluish pigments (Figure S1) [44].

#### 3.1.5. PCA Analysis

**Figure 1:**
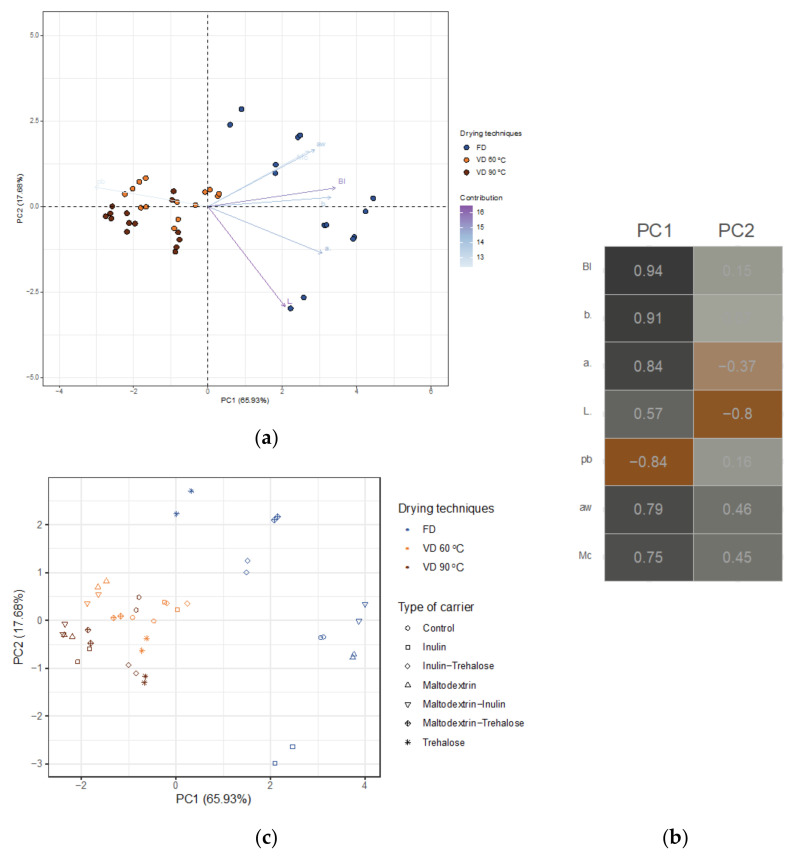
(**a**) The PCA biplot of the first two principal components that simultaneously shows PC scores of chokeberry pomace extract powders (points) and loadings of explanatory variables (vectors). The marker colour corresponds to the drying techniques (i.e., freeze and vacuum drying at 60 and 90 °C), while the length and the transparency of the arrows indicate the variance of the physical properties of powders from chokeberry pomace extracts and their contributions to the principal components, respectively. Together, the first two principal components explain 83.61% of the variability; (**b**) The plot of normalised factor loadings that quantify the extent to which the explanatory variable is related with a given principal component. Applying the so-called Malinowski rule (i.e., normalised factor loading cut-off of |0.70|) simplifies assigning physical meaning to each principal component; (**c**) Score plot in the space defined by the first two principal components illustrating relations and trends of chokeberry pomace extract powders gained after freeze and vacuum drying at 60 and 90 °C with the addition of maltodextrin (M), inulin (I), trehalose (T), maltodextrin—inulin (M—I), maltodextrin—trehalose (M—T), and inulin—trehalose (I—T).

The PCA biplot (Figure 1a) shows that freeze-dried samples had a greater spread and more variance than vacuum-dried samples (at 60 and 90 °C).

The first principal component (PC1) clearly separates freeze-dried samples (positive scores) from vacuum-dried samples (negative scores). The explanatory variables (vectors) with the greatest influence on the separation of chokeberry pomace extract powders in PC1 were the colour parameters, including the browning index (BI), and coordinate a*, b* as well as *Mc*, *a_w_* (positively correlated), and ρ_b_ (negatively correlated). PC2 loadings showed that negatively correlated L* has the greatest influence on the sample distinctions (Figure 1b). Considering the locations of the samples in the space defined by the first two principal components (PCs), it can be stated that, due to low PC1 scores and positive loading values, vacuum-dried samples (at 60 and 90 °C) were characterised by a relatively low browning index (BI), and coordinate a*, b*, as well as *Mc*, *a_w_*. At the same time, powders gained after vacuum drying had the highest values of bulk density. Interestingly, although the type of carrier was also concerned, no straightforward trends were observed for the powders gained (Figure 1c).

### 3.2. Chemical Properties

#### 3.2.1. Polyphenols Content

In the powders obtained from chokeberry pomace extracts, three major groups of polyphenolic compounds were identified, i.e., phenolic acids (3), anthocyanins (4), and flavonols (8). The extraction of chokeberry pomace and usage of absorber technology led to modification of the polyphenolics composition [45] as proanthocyanidins were not identified in the powders obtained. This may be connected with the results reported by Sójka et al. [46] of major absorption of proanthocyanidins in the cell wall of chokeberry pomace. The extraction procedure applied and clarification of the polyphenols by Amberlite 16 of pomace extracts led to an absence of these constituents in the powders. As previously reported by Wang et al. [18], this could be linked to the different affinity of the particular groups of polyphenolics for the stationary phase, which might affect the elution time and thus the presence or absence of these constituents in the extract. Besides this, the presence of phenolic acids, anthocyanins, and flavonols was confirmed [46].

**Figure 2:**
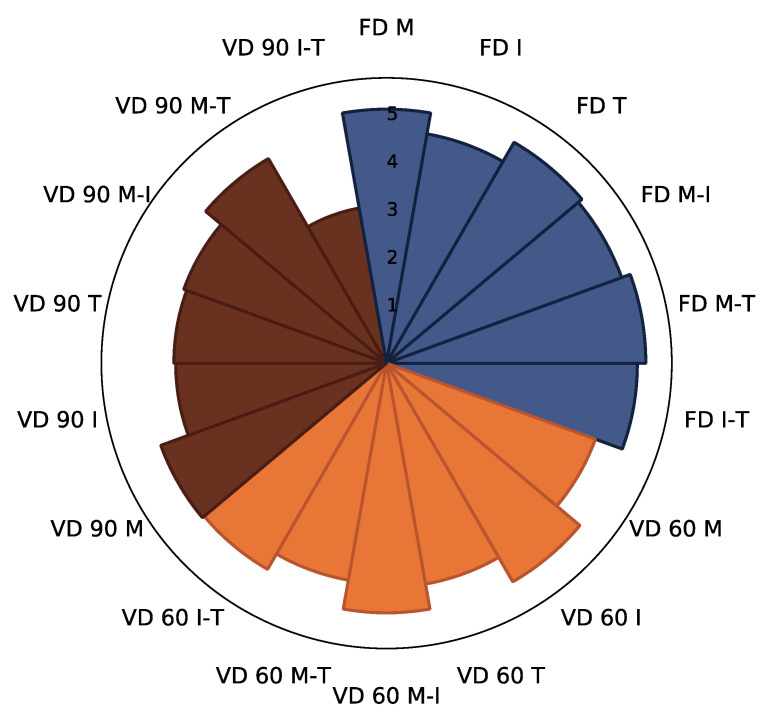
The radar plot of sum of identified polyphenolic compounds in chokeberry pomace extract powders gained with addition of maltodextrin (M), inulin (I), trehalose (T), maltodextrin—inulin (M—I), maltodextrin—trehalose (M—T) and inulin—trehalose (I—T) by freeze drying (FD) and vacuum drying at 60 °C (VD 60) and 90 °C (VD 90) (*n* = 2) (g/100 g db).

The sum of identified polyphenols was, on average, 3.9-fold higher for control samples (Table 2) (average 19.07 g/100 g db) when compared to powders gained with 10% (*w*/*w*) carrier addition to the extracts before drying, regardless of the drying technique applied (Figure 2; Table S1). A similar observation was previously noted in the case of cranberry juices and extracts [47]. The drying techniques influenced the sum of identified polyphenols to a high extent. In the case of control samples, the highest content of polyphenols was noted after freeze drying, followed by vacuum drying at 90 and 60 °C (Table 2). 

In the case of powders produced with the addition of carriers and their mixes, no statistical differences were noted between the average content of polyphenols in the samples gained with the addition of carriers after freeze drying and vacuum drying at 60 °C (Table S1). The strongest influence was noted after the application of vacuum drying at 90 °C. Going into the details the application of the freeze drying process resulted in the highest retention of all identified polyphenols when maltodextrin and the mix of maltodextrin and trehalose (M-T) was used, whereas the usage of inulin resulted in the lowest retention of these constituents (Figure 2); however, the results were not statistically significantly different. One probable cause may be interactions between the carrier and polyphenolics as raised by Tomas et al. [48].

The reverse effect was noted in the case of powders gained after the application of vacuum drying at 60 °C: the addition of inulin resulted in the highest retention of these constituents. Among the powders gained after vacuum drying at 90 °C, the highest retention of the sum of polyphenolics was indicated in the samples obtained after the addition of M and M-T (Figure 2). A statistically significantly lower content of polyphenols was noted when I-T was applied for drying (Table S1). Previously, an influence on the retention of polyphenolics during application of different drying techniques and selected types of carriers was noted for blackcurrant [14] and chokeberry [12] juices.

Going into the details, the percentage share of phenolic acids, anthocyanins, and flavonols in the powders obtained by the selected drying techniques was as follows: 42.9%, 41.2%, and 15.9% for freeze-dried samples; 44%, 37.8%, and 18.2% for vacuum-dried samples at 60 °C; and 47.9%, 31.1%, and 21% for vacuum-dried samples at 90 °C. In order to follow these changes, each group of polyphenolics was examined (Table S2).

**Figure 3:**
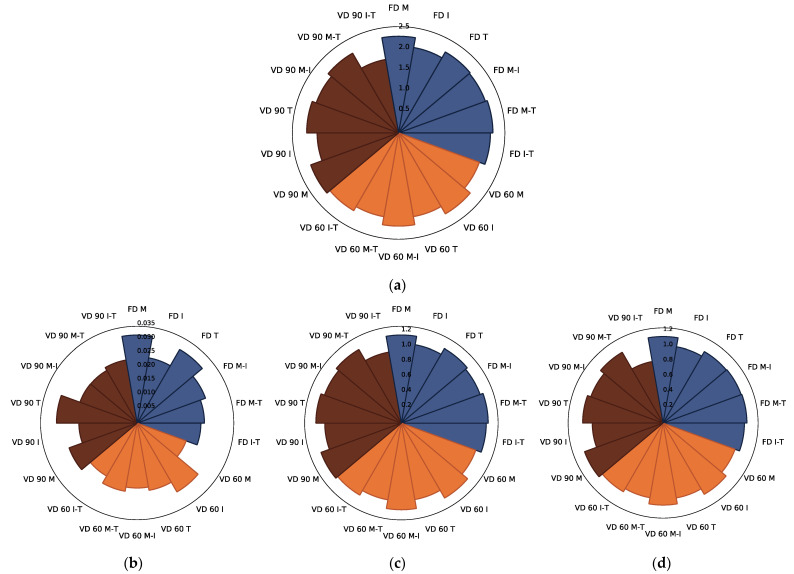
The radar plot of identified phenolic acids ((**a**)—sum of phenolic acids, (**b**)—chlorogenic acid, (**c**)—neochlorogenic acid, (**d**)—cryptochlorogenic acid) in chokeberry pomace extract powders gained with the addition of maltodextrin (M), inulin (I), trehalose (T), maltodextrin—inulin (M—I), maltodextrin—trehalose (M—T) and inulin—trehalose (I—T) by freeze drying (FD) and vacuum drying at 60 (VD 60) and 90 °C (VD 90) (*n* = 2) (g/100 g db).

Similar to chokeberry juice powders [12], the dominant identified group of polyphenolics present in the controls and powders made with the addition of carriers consisted of phenolic acids [49], among which chlorogenic (50.9% of total phenolic acids), neochlorogenic (47.9% of total phenolic acids), and cryptochlorogenic (1.2% of total phenolic acids) acids were quantified (Table 2 and Table S2). In comparison, Sójka et al. [46] identified only chlorogenic and neochlorogenic acids in chokeberry pomace dried at 70 °C. In the current study, the content of phenolic acids was the highest in the control samples gained after freeze and vacuum drying at 90 °C, whereas the application of VD at 60 °C resulted, on average, in a 33% lower content of these constituents (Table 2). When the addition of selected carriers was concerned, the average content of the sum of phenolic acids was at a similar level, regardless of the drying technique and parameters applied (Figure 3a–d). There were no statistically significant differences noted between samples gained with the addition of M, I, T, M-I, M-T, and I-T after freeze and vacuum drying at 60 °C. Vacuum drying at 90 °C caused significant changes in the content of phenolic acids (Table S2). The lowest content of these constituents was noted when I and I-T were added for drying (Figure 3a). A similar observation was noted in the case of chokeberry juice drying, in which addition of inulin resulted in the lowest content of phenolic acids in powders [12]. When the single compounds were concerned (Figure 3b–d), the chlorogenic and neochlorogenic acids followed the comparable alterations caused by the carrier type and drying technique applied. The strongest changes were noted in the case of cryptochlorogenic acid, the content of which was the lowest. Maltodextrin and trehalose preserved the greatest content of this constituent after freeze drying and vacuum drying at 90 °C; however, the latest technique led to the strongest degradation of this compound in the powders gained. Regardless of the quantity of the selected phenolic acids present in the products gained, their thermolability, moderated by the carrier type due to their chemical structure, might be significantly different.

Overall, similar to Tkacz et al. [41], when the type of carrier was concerned, the highest retention of phenolic acids was noted in products gained with maltodextrin in this particular setting of drying techniques. It can be stated that the selection of an appropriate carrier type and drying technique used for the possible highest retention of phenolic acids in plant powders should be tested for specific products (including the initial chemical composition of raw materials) as it may differ due to the interactions between carriers and individual bioactive compounds present in plants [50].

The second group of polyphenolics identified in chokeberry pomace extract powders consisted of anthocyanins, among which the presence of cyanidin-3-*O*-galactoside, -glucoside, -arabinoside, and -xyloside was confirmed (Table 2 and Table 3). Among the controls, freeze drying led to the highest retention of the sum of these constituents followed by VD at 90 and 60 °C (Table 2). A similar observation was made in the case of formulations with carriers, with some exceptions. When inulin was added to the chokeberry extracts, the application of FD and VD at 60 °C resulted in a similar content of identified anthocyanins, whereas the usage of maltodextrin and mix composed of maltodextrin and trehalose resulted in a similar retention of these compounds in the products gained after vacuum drying at 60 and 90 °C. The strongest degradation of these constituents was indicated after vacuum drying at 90 °C for the formulation containing the I-T mixture.

Going into the details, the dominant anthocyanin present in chokeberry pomace extracts’ powders was cyanidin-3-*O*-galactoside, which consisted, on average, of 64.5% of the sum of identified anthocyanins present in powders (controls, carrier added powders), followed by cyanidin-3-*O*-arabinoside, cyanidin-3-*O*-xyloside, and cyanidin-3-*O*-glucoside. A similar percentage share of anthocyanins was previously noted in the case of chokeberry juice powders [12]. What is more, the influence of the carriers used in the study on individual anthocyanins followed the path of the sum of anthocyanins (Table 3).

**Figure 4:**
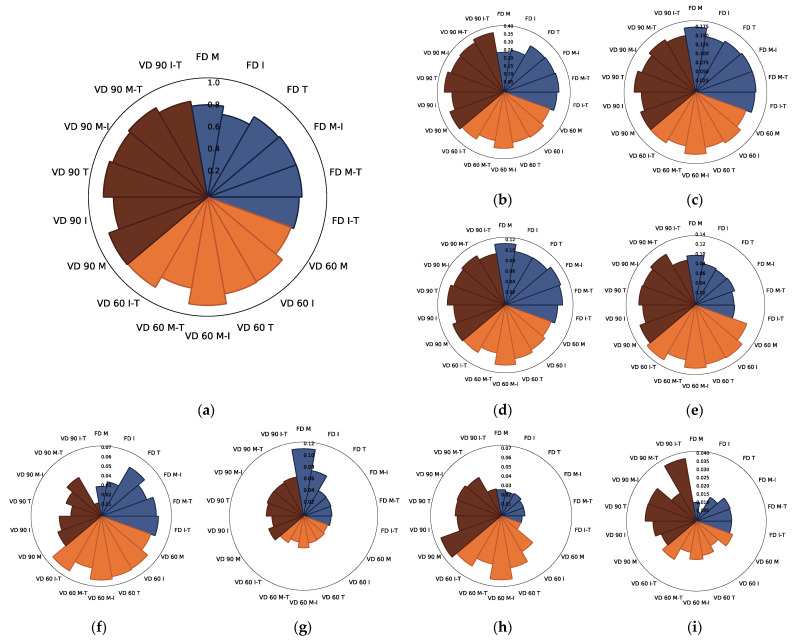
The radar plot of identified flavonols ((**a**)—sum of flavonols, (**b**)—kaempferol-3-*O*-galactoside, (**c**)—kaempferol-3-*O*-glucoside, (**d**)—kaempferol-3-*O*-rutinoside, (**e**)—kaempferol-3-*O*-robinobioside, (**f**)—quercetin-3-*O*-vicianoside, (**g**)—quercetin-dihexoside 1, (**h**)—quercetin-dihexoside 2, (**i**)—derivative of quercetin) in chokeberry pomace extract powders gained with addition of maltodextrin (M), inulin (I), trehalose (T), maltodextrin—inulin (M—I), maltodextrin—trehalose (M—T) and inulin—trehalose (I—T) by freeze drying (FD) and vacuum drying at 60 (VD 60) and 90 °C (VD 90) (*n* = 2) (g/100 g db).

The last of the identified groups of polyphenols in all chokeberry pomace extract powders were flavonols [12] (about 18.3% of all determined compounds). Among them, eight constituents were detected: kaempferol-3-*O*-galactoside, kaempferol-3-*O*-glucoside, kaempferol-3-*O*-rutinoside, kaempferol-3-*O*-robinobioside, quercetin-dihexoside 1, quercetin-3-*O*-vicianoside, quercetin-dihexoside 2, and a derivative of quercetin (Table S3). In comparison with the other groups of polyphenols, the sum of flavonols had the smallest fluctuations in their content depending on the drying technique or carrier type (no statistically significant differences), which confirmed their high stability during the powdering process (Figure 4a; Table S3). However, going into detail, it should be noted that their content was lower in powders obtained by freeze drying (15.9% of all identified polyphenols), while the highest content was reported in products obtained by vacuum drying at 90 °C (21% of all identified polyphenols) (Table S3). As indicated by Hamrouni-Sellami et al. [51], this may be due to the release of these compounds from more polymerised structures during heating. A similar trend was observed for quercetin and its glucoside. In this case, heating up to 120 °C resulted in an increase in their content, while further processing up to 150 °C caused their degradation [52]. However, it is worth looking at the changes in individual flavonols depending on the drying technique used and the addition of the carrier (Figure 4a–i).

The highest content of the dominant flavonol, i.e., kaempferol-3-*O*-galactoside, in the control powders was noted after VD at 60 °C, while the lowest when the FD and VD at 90 °C were applied (Table 2). Considering the powders with carrier addition (Figure 4b), vacuum drying at 90 °C and inulin-trehalose, trehalose, and maltodextrin application was the most suitable, while freeze-dried powders with M and I resulted in the lowest content of this compound, for which statistically significant differences were found (Table S3). 

A very similar relationship was observed for the quercetin derivative in the control samples (Table 2); however, in the case of the carrier-added powders, the lowest content of this constituent was found in freeze-dried products with inulin, while an approximately 7-times higher content was determined in those obtained by the vacuum drying at 90 °C with the addition of I-T mixture (Figure 4i). Interestingly, the parameters and carriers mixture, on the one hand, allowed the highest quercetin derivative content, and on the other hand, resulted in the lowest quercetin-3-*O*-vicianoside concentration (Figure 4f). Comparing the drying techniques, VD at 60 °C was the most favourable in the context of the quercetin-3-*O*-vicianoside level in the powders analysed. Upon comparison of the content of this compound in powders without a carrier, FD and VD at 60 °C proved to be the best methods, while vacuum drying at 90 °C resulted in an approximately 2 times lower content of quercetin-3-*O*-vicianoside (Table 2). Noteworthy, kaempferol-3-*O*-glucoside and kaempferol-3-*O*-rutinoside proved to be the most stable flavonols as their content changed depending on the drying method and the type of added carrier were relatively small compared to the other compounds of the same group (Figure 4c,d). Additionally, fluctuations in the concentration of these compounds depending on the drying parameters and media type were comparable. For kaempferol-3-*O*-robinobioside in the control samples, the lowest concentration of this compound was found in vacuum-dried powders at 60 °C, while FD and VD at 90 °C yielded the highest levels (no statistically significant differences) of this flavonol (Table 2). A reverse effect was observed in the case of powders with carriers (Figure 4e), for which the lowest content of kaempferol-3-*O*-robinobioside was determined in freeze-dried products, while vacuum drying at 60 and 90 °C resulted in the highest and similar content of this constituent in the analysed powders. Taking into account the effect of the carrier agent, the highest content of this compound was found in vacuum-dried powders at 60 °C with I, M-I, and I-T, while the lowest content was found in freeze-dried products with M-T. An interesting relationship was also observed for the two quercetin-dihexosides. In case of quercetin-dihexoside 1 for no-carrier added samples, VD at 60 °C resulted in powders with the lowest concentration of this compound being obtained, while VD at 90 °C resulted in the highest concentration (Table 2). When analysing the powders with added carriers (Figure 4g), the quercetin-dihexoside 1 content was the highest in powders obtained by FD with the addition of M, while considering other drying methods, the same carrier did not have such a noticeable effect as in the case of freeze drying. Moreover, except for the samples with added maltodextrin obtained by FD, no statistically significant differences were found for all the rest of the powders (Table S3). Regarding quercetin-dihexoside 2 for the control powders, no statistically significant differences were found for the content of this compound (Table 2). Interestingly, in the case of powders with added carriers (Figure 4h), freeze drying, irrespective of the type of carrier, as well as vacuum drying at 90 °C and the addition of I-T mixture and VD at 60 °C with M resulted in the lowest content of this compound, while the other variants of powders were characterised by significantly higher quercetin-dihexoside 2 levels (Table S3). Moreover, while for quercetin-dihexoside 1, vacuum drying at 60 °C resulted in its lowest content in the powders obtained, for quercetin-dihexoside 2, the same drying allowed products with the highest level of this compound. Therefore, it can be concluded that the highest quercetin-dihexoside 1 content is possible to obtain if low-temperature drying is used, while the use of high temperatures during drying favours a high quercetin-dihexoside 2 content.

#### 3.2.2. Hydroxymethyl-*L*-furfural

**Figure 5:**
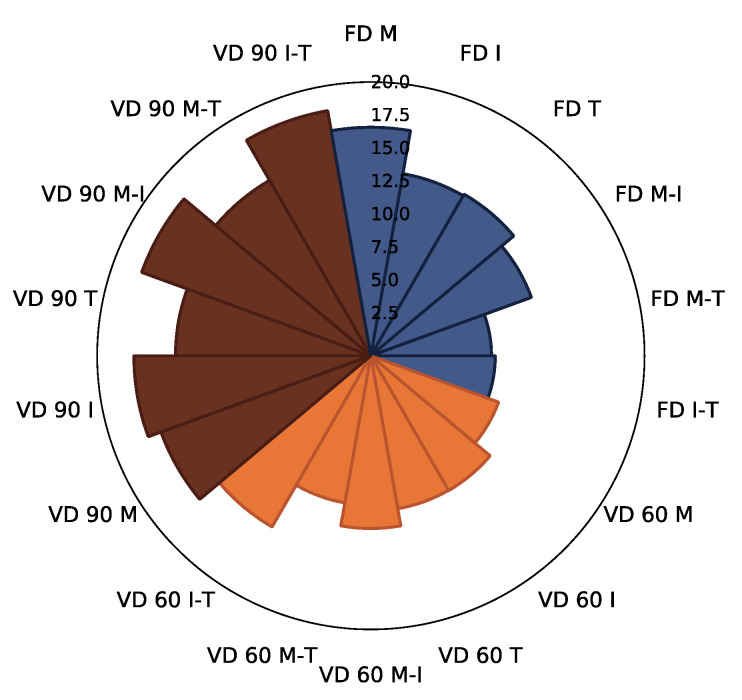
The radar plot of HMF in chokeberry pomace extract powders gained with the addition of maltodextrin (M), inulin (I), trehalose (T), maltodextrin—inulin (M-I), maltodextrin—trehalose (M-T) and inulin—trehalose (I-T) by freeze drying (FD) and vacuum drying at 60 (VD 60) and 90 °C (VD 90) (*n* = 2) (μg/100 g db).

In the current study, the hydroxymethyl-*L*-furfural was identified in all analysed powders (Table 2, Figure 5). In the case of control samples, the highest content of HMF was noted in powders gained after VD at 90 °C and freeze drying (no statistically significant differences) while, interestingly, a lower content was noted after the application of vacuum drying at 60 °C (Table 2). Contrary to the expectations that freeze drying as a low-temperature treatment should result in the lowest content of HMF (or its absence), its presence in the analysed powders might be connected with the particular composition of chokeberry pomace extracts. The substrates for the HMF formation present in these extracts might additionally react with bioactives during the storage of fruit products [53] and even after freeze drying. As a confirmation of this, its occurrence was previously noted in freeze-dried products [54] and fruit juice-based foodstuffs [12,41]. Interestingly, the studies of Zhang et al. [55] showed that the formation of HMF was accelerated by the chlorogenic acid in model systems, which is in accordance with the present study as a strong correlation between HMF and phenolic acids (*r* = 0.84) was noted. What is more, Zhang and An [56] proved that its formation might be inhibited by the interactions with flavonols, which was in line with the present research where the sum of these identified compounds was concerned (*r* = −0.85) (Figure S2). However, this inhibition mechanism of single flavonols is ambiguous as between HMF and two dominant constituents, namely, kaempferol-3-*O*-galactoside and kaempferol-3-*O*-glucoside, strong negative (*r* = −0.97) and positive (*r* = 0.86) correlations, respectively, were observed at the same time (Figure S2).

Similar to the control samples, the strongest formation of HMF was also noted for powders obtained with carriers gained after VD at 90 °C, followed by freeze drying. Its content was the lowest when VD at 60 °C was used (Figure 5). Its slower formation at 60 °C was also reported by Olivares-Tenorio et al. [57] and Michalska et al. [58] during the drying of fruit-based products, whereas a further increase in drying temperature caused its rapid formation. As it was previously reported, such phenomena might be linked to the formation of intermediary compounds during the application of 60 °C [57]. When the type of carrier was considered, the highest content of HMF was noted when inulin and its mixes (I-T, I-M) were used for powder production at VD at 90 °C. Thus, the inulin might enhance the formation of this process contaminant during powder preparation [12]. As the different bioactives might influence the formation of HMF during drying, in the case of powders gained with the addition of carriers, no significant correlation between the sum of identified polyphenols and HMF was noted, with the exception of quercetin-3-*O*-vicianoside (*r* = −0.65) (Figure S3). Probably, as stated by Zhang and An [56], its formation might be inhibited by the interactions with some flavonols; however, until now, no particular compounds were indicated. To sum up, when the quality of chokeberry extract powders is considered, the formation of HMF could be controlled by the initial composition of material submitted for drying, process parameters, and type of carrier used for the powder preparation.

#### 3.2.3. Antioxidant Capacity

**Figure 6:**
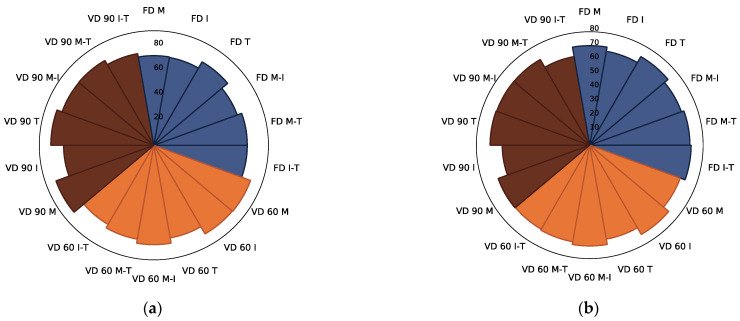
The radar plot of TEAC ABTS (**a**) and FRAP (**b**) of chokeberry pomace extract powders gained with the addition of maltodextrin (M), inulin (I), trehalose (T), maltodextrin—inulin (M—I), maltodextrin—trehalose (M—T), and inulin—trehalose (I—T) by freeze drying (FD) and vacuum drying at 60 (VD 60) and 90 °C (VD 90) (*n* = 2) (mmol Trolox/100 g db).

The antioxidant capacity of chokeberry pomace extract powders determined by the TEAC ABTS and FRAP methods showed that the ability of control samples (Table 2) to scavenge free radicals was about 4.7 times (TEAC ABTS assay) and about 4.2 times higher (FRAP assay) when compared to the powders gained with the application of selected carriers. Going into detail, the phenolic acids significantly influenced the antioxidant capacity measured by TEAC ABTS (*r* = 0.56) and FRAP (*r* = 0.64) in samples produced without the addition of carriers. What is more, a negative correlation between FRAP and the sum of identified flavonols in these products was noticed (*r* = −0.75), which could be connected with the different actions of these compounds present in the analysed powders toward the reducing potential of ferric ion (Fe^3+^ to Fe^2+^) [59]. In the case of the products with added carriers, it was observed that the antioxidant capacity varied slightly depending on the carrier used (Figure 6a,b).

Interestingly, the highest average antioxidant capacity values measured by the TEAC ABTS method were observed in the powders obtained by VD at 90 °C, followed by VD at 60 °C, and freeze drying (Table S1). The reverse effect was observed in the case of FRAP. The antioxidant capacity values may be related to a significant content of certain polyphenolic compounds in those powders, such as predominant phenolic acids [60]. This was confirmed by a high positive correlation (*r* = 0.78) between the content of these constituents in the analysed powders and their antioxidant capacity measured by the FRAP method (Figure S3). Moreover, there was a moderate positive correlation between the sum of anthocyanins and antioxidant capacity measured by FRAP (*r* = 0.58) and no linear relationship between the content of these compounds and TEAC ABTS analysis values (*r* = −0.13) (Figure S3). It might be connected with the lower content of anthocyanins than phenolic acids in the carrier-added powders; however, in the literature, there are also some reports indicating that the antioxidant capacity is related more to the total content of polyphenolic compounds than to the content of anthocyanins, which may be due to the lower free radical scavenging capacity of these compounds compared to other polyphenolics [61]. Furthermore, in the case of TEAC ABTS, and FRAP analysis, the powders obtained with the addition of trehalose and its mixes after freeze drying had the highest values. In general, during VD at 60 °C, the application of inulin resulted in higher TEAC ABTS and FRAP values of the powders analysed, whereas the addition of this carrier and its mix with trehalose lowered the antioxidant properties measured by these two methods when vacuum drying at 90 °C was applied. This proved the selectivity of the action of individual compounds and/or carrier substances [62] toward the free radical scavenging properties of products, which could be additionally moderated by the drying parameters [50].

#### 3.2.4. PCA Analysis

**Figure 7:**
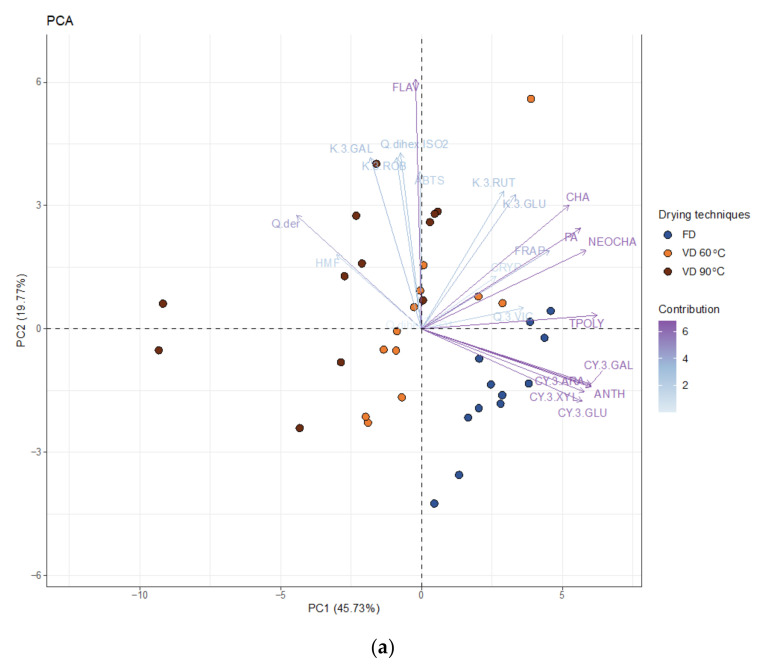

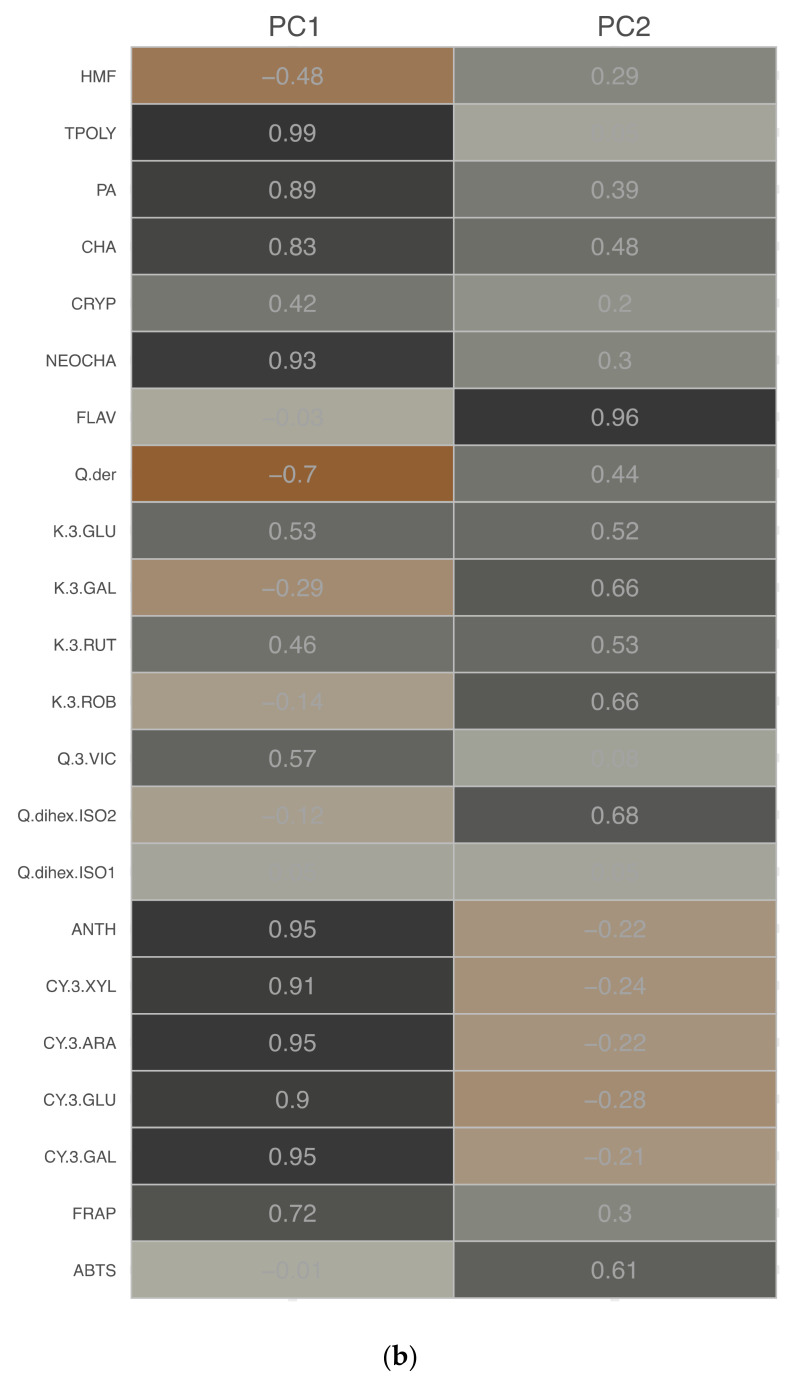

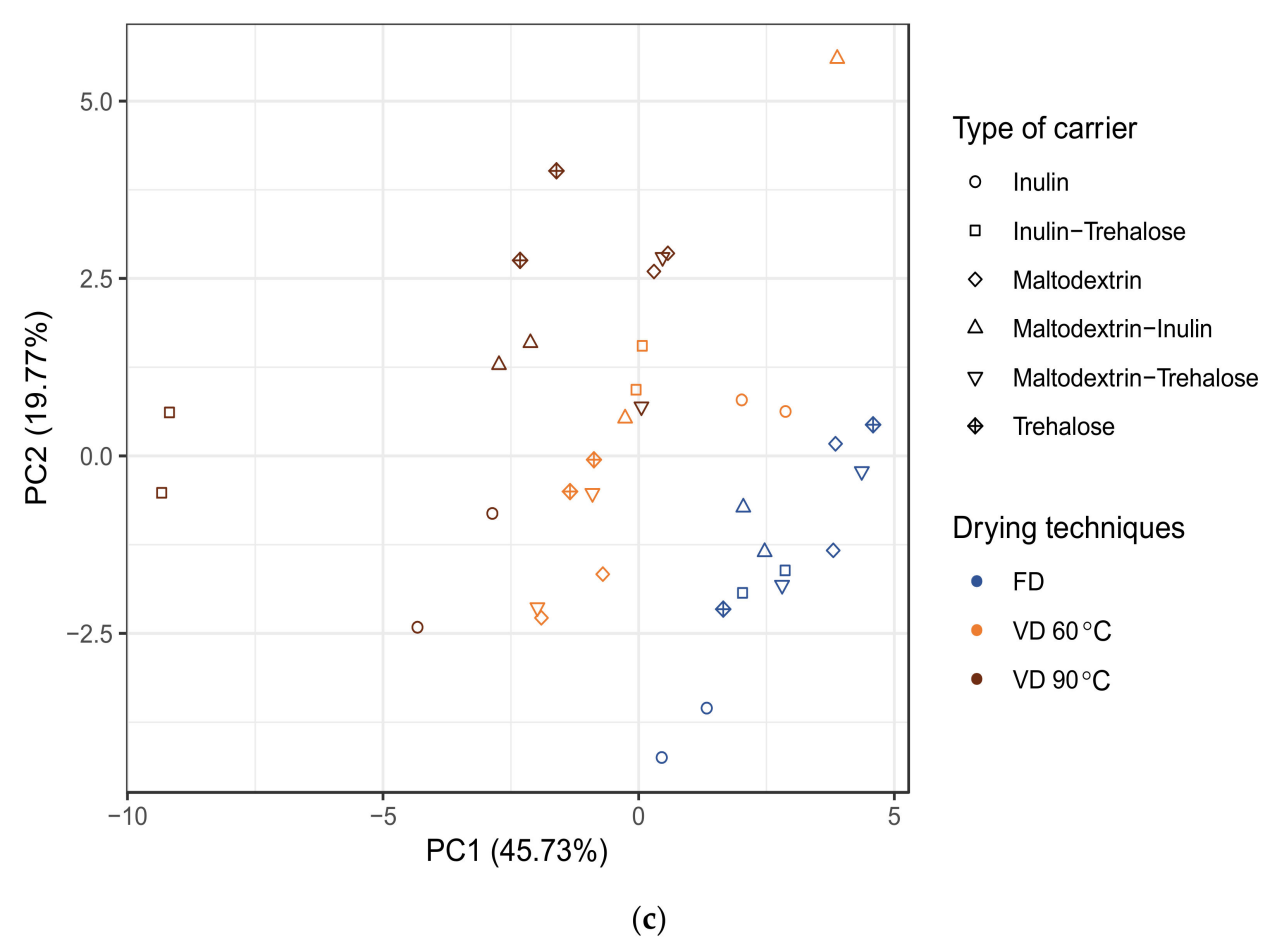
(**a**) The PCA biplot of the first two principal components. The marker colour corresponds to the drying techniques (i.e., freeze and vacuum drying at 60 and 90 °C), while the length and the transparency of the arrows indicate the variance of the chemical properties of powders from chokeberry pomace extracts and their contributions to the principal components, respectively. Together, the first two principal components explain 65.50% of the variability; (**b**) The plot of normalised factor loadings; (**c**) Score plot in the space defined by the first two principal components illustrating the relations and trends of the chokeberry pomace extract powders gained after freeze and vacuum drying at 60 and 90 °C with the addition of maltodextrin (M), inulin (I), trehalose (T), maltodextrin—inulin (M—I), maltodextrin—trehalose (M—T), and inulin—trehalose (I—T).

The PCA biplot for chemical properties (Figure 7a) showed quite the opposite results from those shown for physical properties (Figure 1a).

The vacuum-dried samples at 90 °C had much greater variance than the freeze-dried samples. Chokeberry pomace extract powders gained after the application of different drying techniques were split along PC1. PC1 was positively correlated with the sum of identified polyphenolics (TPOLY), phenolic acids (PA), including chlorogenic acid (CHA), neochlorogenic acid (NEOCHA), the sum of identified anthocyanins (ANTH), including cyanidin-3-*O*-xyloside (CY.3.XYL), -arabinoside (CY.3.ARA), -glucoside (CY.3.GLU), and -galactoside (CY.3.GAL) (Figure 7b). Therefore, it was straightforward to see that drying techniques changed from VD at 90 °C through freeze drying with an increasing content of anthocyanins and phenolic acids, when moving from left to right along the *X*-axis (PC1). PC1 was also negatively correlated with the derivative of quercetin (Q.der). Due to low PC1 scores and negative loading values, samples vacuum dried at 90 °C were characterised by a relatively higher value of Q.der compared to the freeze-dried samples. The most influential variable in PC2 was positively correlated with the sum of flavonols (FLAV). PC2 did not indicate variation, which clearly distinguished between the samples gained after the application of different drying techniques. Hence, only a general statement could be made that chokeberry pomace extract powders toward the top of the PCA biplot (Figure 7a) were described by the highest content of flavonols due to the positive correlations between PC2 and FLAV. A closer look at the score plot shown in Figure 7c revealed that no clear trends were observed for the powders produced with the addition of different carriers and their mixes. Nonetheless, chokeberry pomace extract powders gained after vacuum drying at 90 °C with addition of I-T differed significantly from the rest of the samples in their low content of anthocyanins and phenolic acids.

## 4. Conclusions

The current study evaluated the possibility of obtaining powders from chokeberry pomace extracts by drying techniques and different carrier types as one waste management practice in the food industry. The quality of such products should consider the priorities of their potential application as the moderation of powder properties is a multifactor issue. Taking the above into consideration, the PCA analysis indicated that freeze-dried samples exhibit more variation than those produced by vacuum drying at 60 and 90 °C, especially in terms of moisture content, water activity, colour, and browning index. The bulk density was higher for products obtained after vacuum drying. No straightforward trends in physical properties were observed for products that has selected carriers added.

In the analysed powders, three groups of polyphenols were identified and quantified, i.e., phenolic acids (3), anthocyanins (4), and flavonols (8). Drying techniques significantly influenced the polyphenolics in the powders gained with the addition of selected carriers. In general, the application of freeze drying resulted in a higher content of anthocyanins and phenolic acids, while vacuum drying at 90 °C allowed for the obtainment of products with high quantities of flavonols. Where the analysed carriers were concerned, the highest retention of the sum of identified polyphenolics was noted when maltodextrin and its mixture with trehalose were applied for powder production by freeze drying and vacuum drying at 90 °C, whereas during VD at 60 °C, it was inulin and its mixes. In the case of phenolic acids and anthocyanins, a similar observation was made for FD and VD 60 in that maltodextrin and trehalose protect most of the mentioned compounds; however, in case of VD 90, trehalose caused the lowest retention of anthocyanins. Regarding flavonols, this group was characterised by the highest stability during drying, regardless of the carrier type used. A detailed analysis showed very diverse behaviour of the individual compounds with respect to the applied processing parameters, thus making it impossible to identify any specific method of powder production that results in flavonols’ highest retention.

As the content of hydroxymethyl-*L*-furfural is of high importance to monitor in processed foods’ quality, the lowest concentration of this compound was determined in powders gained after vacuum drying at 60 °C, while its highest level was noted after VD at 90 °C. The current study confirmed [12] that the addition of inulin and its mixes during high-temperature treatment (vacuum drying at 90 °C) should be carefully considered as this carrier may influence the formation of HMF in fruit-based products.

To sum up, the retention of polyphenolics and formation of HMF in chokeberry pomace extracts’ powders was affected simultaneously by the initial composition of raw material, carrier type, drying techniques, and parameters applied. Taking all these factors into account, including interactions between the matrix composition during drying, 10% addition of maltodextrin and trehalose mixture for freeze drying and vacuum drying at 90 °C allowed the production of powders with the highest retention of polyphenolic compounds and the lowest HMF level, at the same time. The outcome of the current study supported by the chemometric analyses can provide guidance for further research as well as give directions for work on designing functional foodstuff based on powders from chokeberry pomace extracts.

## Figures and Tables

**Table 1:** Moisture content, water activity, bulk density, colour (CIE L*a*b*), and browning index of chokeberry powders obtained after freeze and vacuum drying (*n* = 2; average ± standard deviation).

Drying Technique	Process Conditions	Carrier	*Mc*	*a_w_*	ρ_b_	Colour			BI (AU)
			(%)	(−)	(g/cm^3^)	L*	a*	b*	
FD	−60 °C/24 °C	(−)	4.38 ± 0.54 ^cdef^	0.34 ± 0.02 ^h^	0.09 ± 0.01 ^a^	24.20 ± 0.11 ^i^	16.14 ± 0.23 ^i^	4.65 ± 0.02 ^m^	0.42 ± 0.01 ^ij^
		Maltodextrin	4.20 ± 0.42 ^cdef^	0.41 ± 0.02 ^i^	0.12 ± 0.01 ^a^	25.78 ± 0.25 ^j^	22.31 ± 0.07 ^j^	4.09 ± 0.01 ^l^	0.43 ± 0.01 ^k^
		Inulin	4.74 ± 1.04 ^def^	0.15 ± 0.01 ^ab^	0.32 ± 0.02 ^bc^	28.59 ± 0.06 ^k^	25.93 ± 0.04 ^k^	2.26 ± 0.04 ^i^	0.42 ± 0.02 ^i^
		Trehalose	7.59 ± 1.05 ^g^	0.29 ± 0.02 ^gh^	0.61 ± 0.01 ^defg^	13.57 ± 0.16 ^a^	12.43 ± 0.19 ^g^	1.53 ± 0.03 ^h^	0.43 ± 0.01 ^jk^
		Maltodextrin-Inulin	6.15 ± 1.25 ^efg^	0.42 ± 0.02 ^i^	0.17 ± 0.01 ^ab^	23.44 ± 0.71 ^i^	22.94 ± 0.07 ^i^	4.35 ± 0.11 ^l^	0.45 ± 0.01 ^l^
		Maltodextrin-Trehalose	6.53 ± 0.01 ^fg^	0.43 ± 0.01 ^i^	0.29 ± 0.01 ^bc^	17.28 ± 0.03 ^bc^	12.69 ± 0.05 ^g^	3.32 ± 0.02 ^k^	0.43 ± 0.01 ^jk^
		Inulin-Trehalose	5.42 ± 0.70 ^efg^	0.31 ± 0.01 ^h^	0.32 ± 0.03 ^bc^	18.27 ± 0.18 ^cde^	12.89 ± 0.33 ^g^	3.37 ± 0.07 ^k^	0.42 ± 0.01 ^ij^
VD	60 °C	(−)	1.12 ± 0.39 ^a^	0.24 ± 0.01 ^def^	0.64 ± 0.05 ^defgh^	18.48 ± 0.36 ^cdef^	9.76 ± 0.87 ^d^	2.46 ± 0.36 ^ij^	0.39 ± 0.01 ^g^
		Maltodextrin	2.75 ± 0.57 ^abcd^	0.23 ± 0.01 ^def^	0.70 ± 0.01 ^efghi^	16.57 ± 0.11 ^b^	7.38 ± 0.09 ^c^	0.49 ± 0.02 ^cde^	0.37 ± 0.02 ^cd^
		Inulin	2.65 ± 0.04 ^abcd^	0.32 ± 0.01 ^h^	0.65 ± 0.06 ^defgh^	18.55 ± 0.54 ^def^	15.51 ± 0.24 ^i^	1.05 ± 0.02 ^g^	0.41 ± 0.01 ^h^
		Trehalose	2.74 ± 1.16 ^abcd^	0.19 ± 0.01 ^bcde^	0.53 ± 0.05 ^d^	20.55 ± 0.12 ^gh^	10.71 ± 0.03 ^ef^	0.81 ± 0.04 ^fg^	0.38 ± 0.01 ^ef^
		Maltodextrin-Inulin	2.89 ± 0.45 ^abcd^	0.21 ± 0.01 ^cdef^	0.75 ± 0.04 ^fghi^	17.93 ± 0.18 ^cd^	6.34 ± 0.15 ^ab^	0.19 ± 0.03 ^ab^	0.35 ± 0.01 ^ab^
		Maltodextrin-Trehalose	2.02 ± 0.42 ^abc^	0.25 ± 0.01 ^fg^	0.60 ± 0.01 ^def^	19.61 ± 0.19 ^fg^	6.12 ± 0.07 ^ab^	0.39 ± 0.04 ^bcd^	0.35 ± 0.01 ^a^
		Inulin-Trehalose	3.91 ± 0.54 ^bcde^	0.32 ± 0.01 ^h^	0.77 ± 0.08 ^hi^	20.29 ± 0.36 ^gh^	13.79 ± 0.18 ^h^	1.41 ± 0.03 ^h^	0.40 ± 0.01 ^g^
	90 °C	(−)	0.98 ± 0.47 ^a^	0.24 ± 0.01 ^efg^	0.55 ± 0.03 ^de^	17.92 ± 0.11 ^cd^	6.07 ± 0.21 ^ab^	2.71 ± 0.06 ^j^	0.38 ± 0.01 ^f^
		Maltodextrin	0.89 ± 0.38 ^a^	0.14 ± 0.01 ^a^	0.65 ± 0.08 ^defgh^	17.97 ± 0.11 ^cd^	5.66 ± 0.01 ^a^	0.26 ± 0.01 ^abc^	0.35 ± 0.01 ^a^
		Inulin	1.37 ± 0.25 ^a^	0.15 ± 0.02 ^ab^	0.76 ± 0.02 ^ghi^	19.31 ± 0.12 ^efg^	10.49 ± 0.22 ^de^	0.02 ± 0.04 ^a^	0.37 ± 0.01 ^de^
		Trehalose	0.47 ± 0.02 ^a^	0.18 ± 0.01 ^abcd^	0.34 ± 0.02 ^c^	21.06 ± 0.18 ^h^	11.44 ± 0.03 ^f^	0.70 ± 0.03 ^ef^	0.38 ± 0.01 ^ef^
		Maltodextrin-Inulin	1.69 ± 0.28 ^ab^	0.14 ± 0.02 ^a^	0.80 ± 0.04 ^hi^	17.55 ± 0.69 ^bcd^	7.50 ± 0.12 ^c^	0.07 ± 0.09 ^a^	0.36 ± 0.02 ^bc^
		Maltodextrin-Trehalose	0.88 ± 0.12 ^a^	0.16 ± 0.02 ^ab^	0.57 ± 0.06 ^de^	18.15 ± 0.13 ^cde^	6.80 ± 0.25 ^bc^	0.52 ± 0.04 ^cde^	0.36 ± 0.01 ^b^
		Inulin-Trehalose	2.62 ± 0.09 ^abcd^	0.17 ± 0.01 ^abc^	0.83 ± 0.01^i^	21.75 ± 0.77 ^h^	15.8 ± 0.27 ^i^	0.67 ± 0.04 ^def^	0.39 ± 0.01 ^g^

(−)—no carrier addition, FD—freeze drying, VD—vacuum drying, *M_C_*—moisture content, *a_w_*—water activity, ρ_b_—bulk density, AU—arbitrary units; ^a–m^ the same letters within a column indicate no statistically significant differences (HSD Tukey test; *p* ≤ 0.05).

**Table 2:** The content of identified polyphenolics (g/100 g db), hydroxymethyl-*L*-furfural (μg/100 g db) and antioxidant capacity measured by TEAC ABTS and FRAP methods (mmol TE/100 g db) in chokeberry pomace extract powders (controls) obtained after freeze and vacuum drying (average ± standard deviation; *n* = 2).

	FD	VD	
	−60 °C/+24 °C	60 °C	90 °C
Total polyphenols	22.70 ± 0.12 ^c^	15.88 ± 0.44 ^a^	18.64 ± 0.47 ^b^
Phenolic acids Neochlorogenic acid Cryptochlorogenic acid Chlorogenic acid Sum			
	4.86 ± 0.18 ^b^	3.01 ± 0.09 ^a^	4.47 ± 0.67 ^ab^
	0.10 ± 0.01 ^ab^	0.07 ± 0.01 ^a^	0.14 ± 0.02 ^b^
	5.15 ± 0.15 ^b^	3.43 ± 0.11 ^a^	4.72 ± 0.37 ^b^
	10.10 ± 0.02 ^b^	6.51 ± 0.20 ^a^	9.33 ± 1.06 ^b^
Anthocyanins			
Cyanidin-3-*O*-galactoside	5.90 ± 0.14 ^b^	3.68 ± 0.09 ^a^	3.93 ± 0.37 ^a^
Cyanidin-3-*O*-glucoside	0.33 ± 0.05 ^b^	0.18 ± 0.01 ^a^	0.22 ± 0.02 ^ab^
Cyanidin-3-*O*-arabinoside	2.56 ± 0.05 ^b^	1.68 ± 0.05 ^a^	1.61 ± 0.13 ^a^
Cyanidin-3-*O*-xyloside	0.41 ± 0.01 ^b^	0.28 ± 0.02 ^a^	0.29 ± 0.03 ^a^
Sum	9.19 ± 0.13 ^b^	5.82 ± 0.12 ^a^	6.06 ± 0.56 ^a^
Flavonols			
Quercetin-dihexoside 1	0.26 ± 0.01 ^b^	0.17 ± 0.01 ^a^	0.30 ± 0.01 ^c^
Quercetin-dihexoside 2	0.14 ± 0.01 ^a^	0.21 ± 0.01 ^a^	0.21 ± 0.04 ^a^
Quercetin-3-*O*-vicianoside	0.14 ± 0.01 ^b^	0.12 ± 0.01 ^b^	0.06 ± 0.02 ^a^
Kaempferol-3-*O*-robinobioside	0.45 ± 0.02 ^b^	0.24 ± 0.01 ^a^	0.43 ± 0.02 ^b^
Kaempferol-3-*O*-rutinoside	0.53 ± 0.02 ^b^	0.30 ± 0.01 ^a^	0.48 ± 0.02 ^b^
Kaempferol-3-*O*-galactoside	1.06 ± 0.02 ^a^	1.72 ± 0.07 ^b^	1.03 ± 0.01 ^a^
Kaempferol-3-*O*-glucoside	0.78 ± 0.02 ^b^	0.59 ± 0.02 ^a^	0.72 ± 0.02 ^b^
Derivative of quercetin	0.05 ± 0.01 ^b^	0.20 ± 0.01 ^c^	0.04 ± 0.01 ^a^
Sum	3.40 ± 0.01 ^a^	3.55 ± 0.12 ^a^	3.26 ± 0.03 ^a^
Hydroxymethyl-*L*-furfural	11.57 ± 0.23 ^a^	2.62 ± 0.24 ^b^	14.03 ± 1.59 ^a^
TEAC ABTS	364.95 ± 11.98 ^a^	357.33 ± 11.01 ^a^	374.80 ± 12.92 ^a^
FRAP	292.95 ± 7.61 ^a^	285.14 ± 1.02 ^a^	299.38 ± 1.01 ^a^

FD—freeze drying, VD—vacuum drying; TE—Trolox equivalent; ^a,b,c^—the same letters within a row indicate no statistically significant differences (HSD Tukey test, *p* ≤ 0.05).

**Table 3:** The content of identified anthocyanins in chokeberry pomace extracts powders (g/100 g db) (*n* = 2; average ± standard deviation).

Drying Technique	Process Conditions	Carrier	Anthocyanins				
			Cyanidin-3-*O*-Galactoside	Cyanidin-3-*O*-Glucoside	Cyanidin-3-*O*-Arabinoside	Cyanidin-3-*O*-Xyloside	Sum of Anthocyanins
FD	−60 °C/24 °C	Maltodextrin	1.36 ± 0.04 ^fg^	0.08 ± 0.01 ^g^	0.59 ± 0.01 ^fgh^	0.10 ± 0.01 ^fg^	2.12 ± 0.06 ^ghi^
		Inulin	1.28 ± 0.06 ^efg^	0.07 ± 0.01 ^defg^	0.56 ± 0.03 ^efgh^	0.10 ± 0.01 ^efg^	2.00 ± 0.10 ^fghi^
		Trehalose	1.42 ± 0.08 ^g^	0.07 ± 0.01 ^efg^	0.62 ± 0.04 ^gh^	0.11 ± 0.01 ^g^	2.22 ± 0.14 ^hi^
		Maltodextrin—Inulin	1.38 ± 0.05 ^g^	0.06 ± 0.01 ^cdefg^	0.59 ± 0.02 ^fgh^	0.10 ± 0.01 ^fg^	2.13 ± 0.07 ^ghi^
		Maltodextrin—Trehalose	1.45 ± 0.02 ^g^	0.07 ± 0.01 ^fg^	0.64 ± 0.02 ^h^	0.11 ± 0.01 ^g^	2.27 ± 0.04 ^i^
		Inulin—Trehalose	1.41 ± 0.04 ^g^	0.07 ± 0.01 ^defg^	0.62 ± 0.02 ^gh^	0.09 ± 0.01 ^defg^	2.18 ± 0.08 ^hi^
VD	60 °C	Maltodextrin	1.13 ± 0.03 ^cde^	0.05 ± 0.01 ^bc^	0.49 ± 0.01 ^de^	0.08 ± 0.01 ^bcd^	1.75 ± 0.05 ^cdef^
		Inulin	1.37 ± 0.06 ^fg^	0.06 ± 0.01 ^cdef^	0.60 ± 0.03 ^fgh^	0.09 ± 0.01 ^efg^	2.12 ± 0.09 ^ghi^
		Trehalose	1.12 ± 0.04 ^cde^	0.05 ± 0.01 ^bc^	0.48 ± 0.02 ^cde^	0.08 ± 0.01 ^cde^	1.73 ± 0.07 ^cdef^
		Maltodextrin—Inulin	1.27 ± 0.10 ^efg^	0.06 ± 0.01 ^cdef^	0.55 ± 0.05 ^efg^	0.09 ± 0.01 ^cdef^	1.97 ± 0.15 ^efgh^
		Maltodextrin—Trehalose	1.10 ± 0.04 ^cde^	0.05 ± 0.01 ^bc^	0.47 ± 0.02 ^cde^	0.07 ± 0.01 ^bc^	1.70 ± 0.07 ^cde^
		Inulin—Trehalose	1.19 ± 0.01 ^def^	0.06 ± 0.01 ^cde^	0.52 ± 0.01 ^ef^	0.09 ± 0.01 ^cdef^	1.85 ± 0.01 ^defg^
	90 °C	Maltodextrin	1.16 ± 0.01 ^de^	0.06 ± 0.01 ^cdef^	0.49 ± 0.01 ^de^	0.08 ± 0.01 ^cdef^	1.80 ± 0.01 ^def^
		Inulin	1.03 ± 0.02 ^cd^	0.05 ± 0.01 ^bc^	0.43 ± 0.01 ^bcd^	0.07 ± 0.01 ^bcd^	1.58 ± 0.04 ^cd^
		Trehalose	0.82 ± 0.02 ^b^	0.04 ± 0.01 ^b^	0.35 ± 0.01 ^b^	0.06 ± 0.01 ^b^	1.27 ± 0.03 ^b^
		Maltodextrin—Inulin	0.95 ± 0.04 ^bc^	0.05 ± 0.01 ^bc^	0.40 ± 0.02 ^bc^	0.07 ± 0.01 ^bc^	1.47 ± 0.07 ^bc^
		Maltodextrin—Trehalose	1.15 ± 0.02 ^de^	0.05 ± 0.01 ^bcd^	0.48 ± 0.01 ^cde^	0.08 ± 0.01 ^cdef^	1.76 ± 0.02 ^def^
		Inulin—Trehalose	0.39 ± 0.01 ^a^	0.02 ± 0.01 ^a^	0.16 ± 0.01 ^a^	0.03 ± 0.01 ^a^	0.60 ± 0.02 ^a^

FD—freeze drying, VD—vacuum drying; ^a–i^—the same letters within a column indicate no statistically significant differences (HSD Tukey test, *p* ≤ 0.05).

## Supplementary Materials

The following are available online at https://www.mdpi.com/article/10.3390/foods10081864/s1, Figure S1: A correlation matrix showing the Pearson’s correlation coefficients between each pair of variables (i.e., physical properties) in chokeberry pomace extract powders data set (controls and powders with carrier addition), Figure S2: A correlation matrix showing the Pearson’s correlation coefficients between each pair of variables (i.e., chemical properties) in chokeberry pomace extract powders data set (control samples), Figure S3: A correlation matrix showing the Pearson’s correlation coefficients between each pair of variables (i.e., chemical properties) in chokeberry pomace extract powders data set (powders with addition of carriers; no controls included), Table S1: The content of sum of polyphenols, hydroxymethyl-*L*-furfural and the antioxidant capacity measured by TEAC ABTS and FRAP methods of chokeberry pomace extracts powders made with the addition maltodextrin, inulin, trehalose and a mixture of them using different drying methods (average ± standard deviation; *n* = 2), Table S2: The content of identified phenolic acids in chokeberry pomace extracts powders made with the addition maltodextrin, inulin, trehalose and a mixture of them using different drying methods (g/100 g db) (*n* = 2; average ± standard deviation), Table S3: The content of identified flavonols in chokeberry pomace extracts powders made with the addition maltodextrin, inulin, trehalose and a mixture of them using different drying methods (g/100 g db) (*n* = 2; average ± standard deviation).
