# Supplementary material for: How Do the Different Types of Carrier and Drying Techniques Affect the Changes in Physico-Chemical Properties of Powders from Chokeberry Pomace Extracts?
Source: Foods. 2021 Aug 12;10(8):1864. doi: 10.3390/foods10081864 (PMC8394545; doi:10.3390/foods10081864)
Supplement: Supplementary file 1 [file foods-10-01864-s001.zip › foods-1289858 - Supplementary Materials.pdf]

Supplementary Materials:

Figures

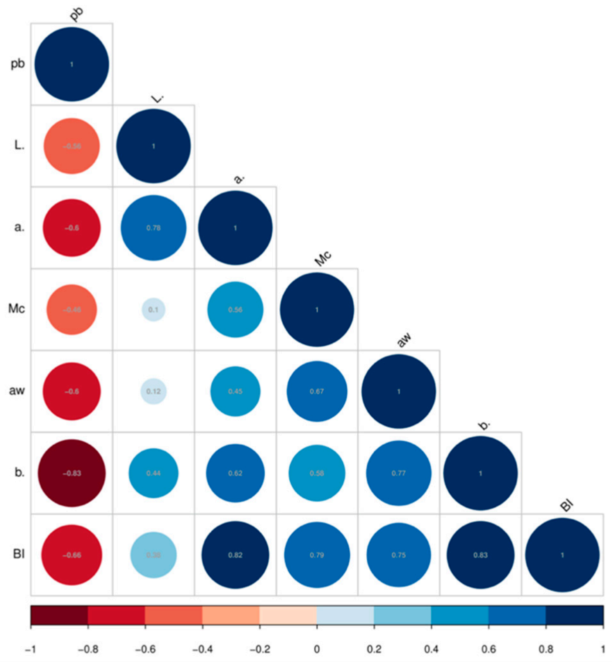

Figure S1. A correlation matrix showing the Pearson's correlation coefficients between each pair of variables (i.e., physical properties) in chokeberry pomace extract powders data set (controls and powders with carrier addition). The shape and colour intensity of the circle/ellipses displayed in the lower triangular part of a correlation matrix are proportional to the strength and direction of a linear relationship between any two variables. The highest correlation coefficients (shown as a thin ellipse) are considered as important, whereas the smallest correlation coefficients that reflect the weak linear relationship between the two variables are considered as insignificant and indicated as big circles. Positive correlations are marked in blue, while negative correlations in red colour. The values of the corresponding correlation coefficients expressed in numerical forms are shown in the upper triangular part of this correlation matrix.

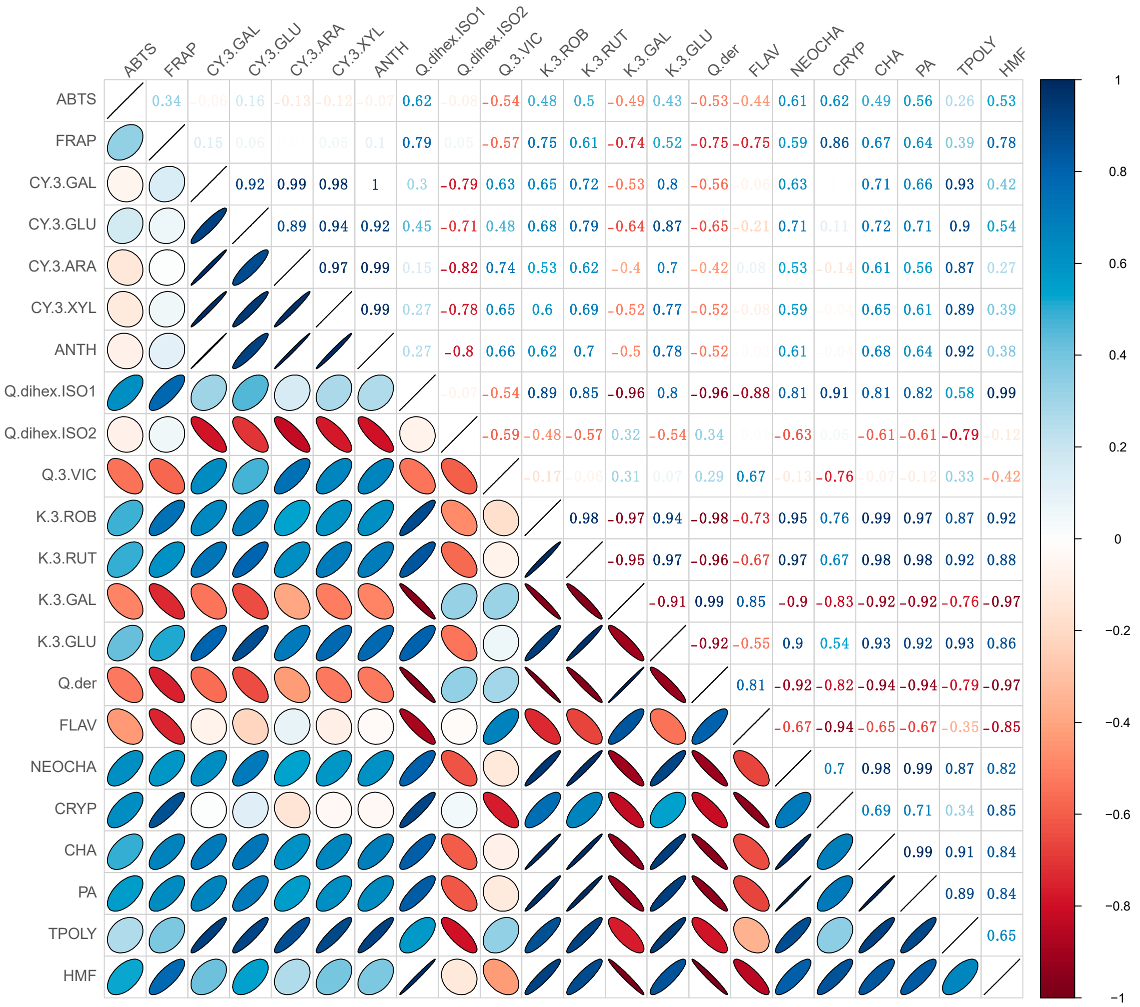

Figure S2. A correlation matrix showing the Pearson's correlation coefficients between each pair of variables (i.e., chemical properties) in chokeberry pomace extract powders data set (control samples).

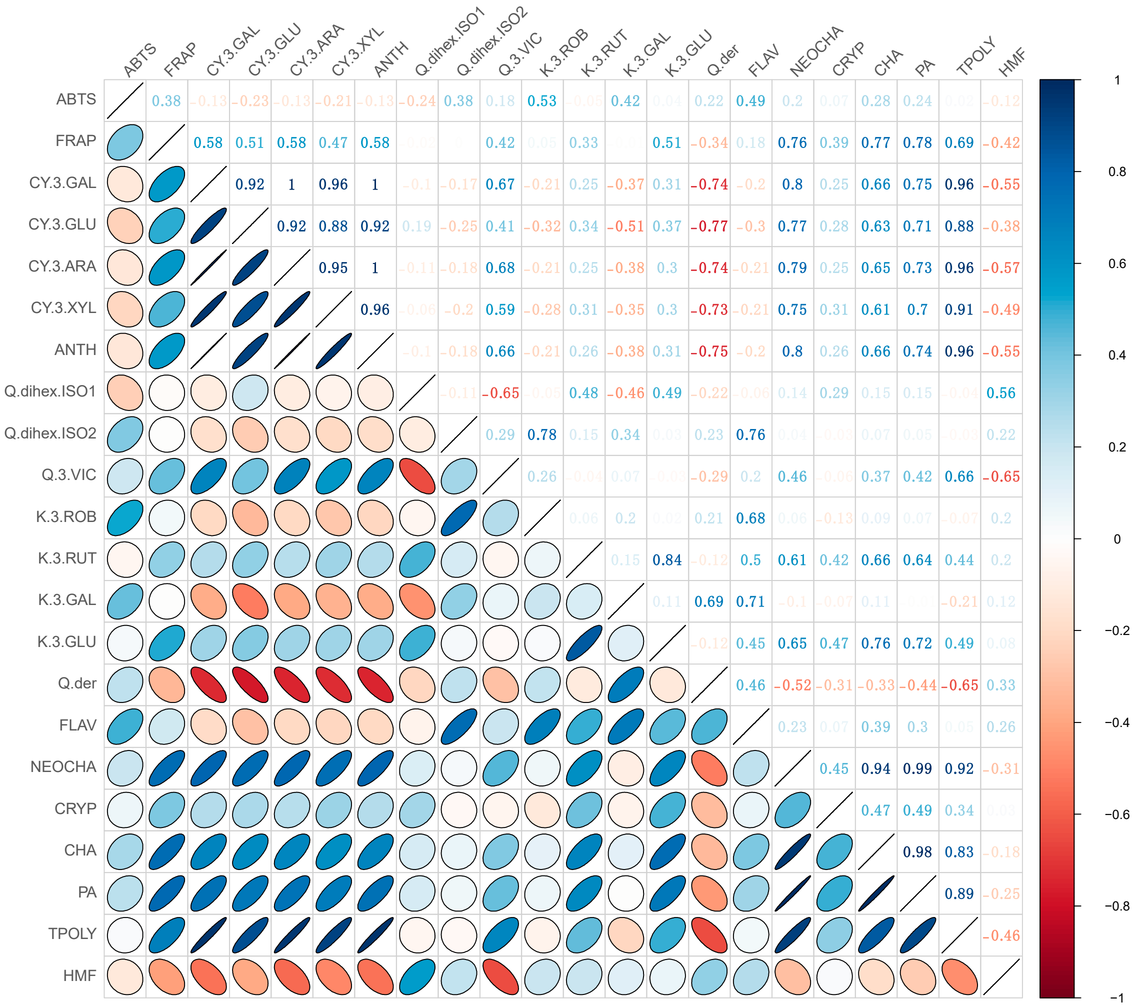

Figure S3. A correlation matrix showing the Pearson's correlation coefficients between each pair of variables (i.e., chemical properties) in chokeberry pomace extract powders data set (powders with addition of carriers; no controls included).

Tables

Table S1. The content of sum of polyphenols, hydroxymethyl-*L*-furfural and the antioxidant capacity measured by TEAC ABTS and FRAP methods of chokeberry pomace extracts powders made with the addition maltodextrin, inulin, trehalose and a mixture of them using different drying methods (average ± standard deviation; *n* = 2).

Drying technique	Process conditions	Carrier	Sum of polyphenols (g/100 g db)	ABTS (mmol Trolox/100 g db)	FRAP (mmol Trolox/100 g db)	HMF (µg/100 g db)
FD	−60 °C / 24 °C	Maltodextrin	5.31 ± 0.81 ^{de}	72.90 ± 2.48 ^{ab}	70.16 ± 1.31 ^{cd}	17.30 ± 2.50 ^{cd}
		Inulin	4.86 ± 0.75 ^{bcde}	72.60 ± 0.92 ^{a}	67.96 ± 1.84 ^{bc}	13.99 ± 1.21 ^{abcd}
		Trehalose	5.33 ± 0.81^{de}	78.36 ± 1.50 ^{bcde}	72.24 ± 1.64 ^{cd}	14.12 ± 0.32 ^{abcd}
		Maltodextrin—Inulin	5.23 ± 0.77 ^{cde}	71.40 ± 2.71 ^{a}	69.25 ± 1.31 ^{cd}	12.93 ± 2.04 ^{abc}
		Maltodextrin—Trehalose	5.42 ± 0.82 ^{e}	76.06 ± 0.65 ^{abcd}	70.30 ± 2.60 ^{cd}	9.14 ± 0.50 ^{a}
		Inulin—Trehalose	5.24 ± 0.79 ^{cde}	76.26 ± 1.30 ^{abcd}	72.12 ± 1.22 ^{cd}	9.43 ± 1.47 ^{a}
VD	60 °C	Maltodextrin	4.64 ± 0.66 ^{bcd}	83.37 ± 0.84 ^{ef}	68.32 ± 0.58 ^{bcd}	10.29 ± 1.35 ^{ab}
		Inulin	5.27 ± 0.76 ^{cde}	83.31 ± 0.62 ^{ef}	73.01 ± 3.01 ^{d}	11.79 ± 2.30 ^{ab}
		Trehalose	4.68 ± 0.60 ^{bcd}	76.46 ± 0.80 ^{abcd}	67.23 ± 1.79 ^{bc}	11.76 ± 0.14 ^{ab}
		Maltodextrin—Inulin	5.21 ± 0.67 ^{cde}	79.91 ± 3.15 ^{def}	72.15 ± 4.03 ^{cd}	13.08 ± 1.60 ^{abc}
		Maltodextrin—Trehalose	4.60 ± 0.63 ^{bc}	77.36 ± 1.78 ^{abcd}	69.36 ± 0.66 ^{cd}	11.32 ± 1.40 ^{ab}
		Inulin—Trehalose	4.97 ± 0.64 ^{bcde}	73.90 ± 2.46 ^{abc}	68.56 ± 1.84 ^{bcd}	14.96 ± 0.56 ^{bcd}
	90 °C	Maltodextrin	5.02 ± 0.67 ^{bcde}	84.42 ± 1.07 ^{f}	69.07 ± 4.47 ^{bcd}	16.91 ± 0.49 ^{cd}
		Inulin	4.41 ± 0.57 ^{b}	73.39 ± 0.54 ^{ab}	62.23 ± 1.98 ^{a}	17.94 ± 0.77 ^{cd}
		Trehalose	4.44 ± 0.67 ^{b}	83.53 ± 2.83 ^{ef}	70.67 ± 0.86 ^{cd}	14.79 ± 0.06 ^{bcd}
		Maltodextrin—Inulin	4.51 ± 0.61 ^{b}	79.38 ± 0.41 ^{cdef}	69.9 ± 1.04 ^{cd}	18.47 ± 0.30 ^{d}
		Maltodextrin—Trehalose	4.93 ± 0.66 ^{bcde}	79.46 ± 0.71 ^{cdef}	70.07 ± 0.92 ^{cd}	15.33 ± 1.15 ^{bcd}
		Inulin—Trehalose	3.29 ± 0.63 ^{a}	75.66 ± 1.05 ^{abcd}	63.70 ± 0.43 ^{ab}	18.84 ± 0.33 ^{d}

FD—freeze drying, VD—vacuum-drying; $^{a-f}$ the same letters within a column indicate no statistically significant differences (HSD Tukey test, $p \leq 0.05$).

Table S2. The content of identified phenolic acids in chokeberry pomace extracts powders made with the addition maltodextrin, inulin, trehalose and a mixture of them using different drying methods (g/100 g db) (n = 2; average ± standard deviation).

Drying technique	Process conditions	Carrier	Phenolic acids			
			**Neochlorogenic acid**	**Cryptochlorogenic acid**	**Chlorogenic acid**	**Sum of phenolic acids**
FD	−60 °C / 24 °C	Maltodextrin	1.14 ± 0.02 ^{c}	0.032 ± 0.003 ^{c}	1.17 ± 0.02 ^{b}	2.34 ± 0.65 ^{c}
		Inulin	1.02 ± 0.01 ^{bc}	0.024 ± 0.004 ^{abc}	1.05 ± 0.02 ^{ab}	2.10 ± 0.59 ^{abc}
		Trehalose	1.09 ± 0.07 ^{c}	0.031 ± 0.004 ^{bc}	1.14 ± 0.07 ^{b}	2.27 ± 0.63 ^{bc}
		Maltodextrin—Inulin	1.10 ± 0.02 ^{c}	0.026 ± 0.001 ^{abc}	1.11 ± 0.03 ^{ab}	2.24 ± 0.62 ^{bc}
		Maltodextrin—Trehalose	1.11 ± 0.04 ^{c}	0.024 ± 0.003 ^{abc}	1.15 ± 0.03 ^{b}	2.29 ± 0.64 ^{c}
		Inulin—Trehalose	1.08 ± 0.01 ^{bc}	0.023 ± 0.003 ^{abc}	1.13 ± 0.01 ^{b}	2.23 ± 0.62 ^{bc}
VD	60 °C	Maltodextrin	1.01 ± 0.02 ^{bc}	0.019 ± 0.003 ^{a}	1.05 ± 0.02 ^{ab}	2.08 ± 0.59 ^{abc}
		Inulin	1.09 ± 0.04 ^{c}	0.029 ± 0.001 ^{abc}	1.15 ± 0.04 ^{b}	2.27 ± 0.63 ^{bc}
		Trehalose	1.01 ± 0.02 ^{bc}	0.025 ± 0.004 ^{abc}	1.03 ± 0.02 ^{ab}	2.06 ± 0.57 ^{abc}
		Maltodextrin—Inulin	1.09 ± 0.11 ^{c}	0.024 ± 0.002 ^{abc}	1.15 ± 0.12 ^{b}	2.26 ± 0.63 ^{bc}
		Maltodextrin—Trehalose	1.00 ± 0.03 ^{bc}	0.025 ± 0.002 ^{abc}	1.03 ± 0.03 ^{ab}	2.06 ± 0.57 ^{abc}
		Inulin—Trehalose	1.05 ± 0.01 ^{bc}	0.022 ± 0.001 ^{abc}	1.10 ± 0.01 ^{ab}	2.18 ± 0.61 ^{bc}
	90 °C	Maltodextrin	1.11 ± 0.01 ^{c}	0.026 ± 0.003 ^{abc}	1.14 ± 0.01 ^{b}	2.27 ± 0.63 ^{bc}
		Inulin	0.94 ± 0.04 ^{ab}	0.021 ± 0.002 ^{ab}	1.02 ± 0.05 ^{ab}	1.98 ± 0.55 ^{ab}
		Trehalose	1.07 ± 0.01 ^{bc}	0.029 ± 0.002 ^{bc}	1.13 ± 0.01 ^{b}	2.23 ± 0.62 ^{bc}
		Maltodextrin—Inulin	1.02 ± 0.02 ^{bc}	0.022 ± 0.001 ^{abc}	1.09 ± 0.01 ^{ab}	2.13 ± 0.60 ^{bc}
		Maltodextrin—Trehalose	1.08 ± 0.02 ^{bc}	0.022 ± 0.001 ^{abc}	1.13 ± 0.03 ^{b}	2.23 ± 0.63 ^{bc}
		Inulin—Trehalose	0.83 ± 0.01 ^{a}	0.023 ± 0.002 ^{abc}	0.96 ± 0.01 ^{a}	1.81 ± 0.51 ^{a}

FD—freeze drying, VD—vacuum drying; $^{a-c}$ the same letters within a column indicate no statistically significant differences (HSD Tukey test, $p \leq 0.05$).

Table S3. The content of identified flavonols in chokeberry pomace extracts powders made with the addition maltodextrin, inulin, trehalose and a mixture of them using different drying methods (g/100 g db) (n = 2; average ± standard deviation).

	Carrier	Flavonols								
		Quercetin-dihexoside 1	Quercetin-dihexoside 2	Quercetin-3-*O*-vicianoside	Kaempferol-3-*O*-robinobioside	Kaempferol-3-*O*-rutinoside	Kaempferol-3-*O*-galactoside	Kaempferol-3-*O*-glucoside	Derivative of quercetin	Sum of flavonols
Freeze Drying	M	0.12 ± 0.01 ^{b}	0.03 ± 0.01 ^{abc}	0.03 ± 0.01 ^{ab}	0.10 ± 0.01 ^{bcd}	0.12 ± 0.01 ^{b}	0.25 ± 0.01 ^{a}	0.18 ± 0.01 ^{b}	0.01 ± 0.01 ^{ab}	0.84 ± 0.08 ^{abc}
	I	0.08 ± 0.04 ^{a}	0.03 ± 0.01 ^{ab}	0.04 ± 0.02 ^{bc}	0.09 ± 0.01 ^{ab}	0.10 ± 0.01 ^{ab}	0.27 ± 0.06 ^{ab}	0.16 ± 0.01 ^{ab}	0.01 ± 0.01 ^{a}	0.76 ± 0.08 ^{a}
	T	0.05 ± 0.01 ^{a}	0.03 ± 0.01 ^{abc}	0.06 ± 0.01 ^{def}	0.08 ± 0.01 ^{ab}	0.10 ± 0.01 ^{ab}	0.34 ± 0.02 ^{bc}	0.16 ± 0.01 ^{ab}	0.02 ± 0.01 ^{bc}	0.84 ± 0.10 ^{abc}
	M–I	0.05 ± 0.01 ^{a}	0.03 ± 0.01 ^{ab}	0.05 ± 0.01 ^{bcdef}	0.09 ± 0.01 ^{ab}	0.11 ± 0.01 ^{ab}	0.34 ± 0.01 ^{bc}	0.17 ± 0.01 ^{ab}	0.02 ± 0.01 ^{cde}	0.85 ± 0.11 ^{abc}
	M–T	0.05 ± 0.01 ^{a}	0.03 ± 0.01 ^{ab}	0.06 ± 0.01 ^{def}	0.08 ± 0.01 ^{a}	0.11 ± 0.01 ^{ab}	0.35 ± 0.01 ^{c}	0.17 ± 0.01 ^{ab}	0.02 ± 0.01 ^{cde}	0.86 ± 0.11 ^{abc}
	I–T	0.05 ± 0.01 ^{a}	0.02 ± 0.01 ^{a}	0.06 ± 0.01 ^{ef}	0.08 ± 0.01 ^{ab}	0.10 ± 0.01 ^{ab}	0.33 ± 0.01 ^{bc}	0.17 ± 0.01 ^{ab}	0.02 ± 0.01 ^{cde}	0.83 ± 0.10 ^{ab}
Vacuum Drying 60 °C	M	0.04 ± 0.01 ^{a}	0.02 ± 0.01 ^{a}	0.06 ± 0.01 ^{cdef}	0.11 ± 0.01 ^{cde}	0.09 ± 0.01 ^{a}	0.31 ± 0.01 ^{abc}	0.15 ± 0.01 ^{a}	0.02 ± 0.01 ^{cde}	0.81 ± 0.09 ^{ab}
	I	0.05 ± 0.01 ^{a}	0.04 ± 0.03 ^{abcd}	0.06 ± 0.01 ^{ef}	0.12 ± 0.01 ^{e}	0.10 ± 0.01 ^{ab}	0.33 ± 0.01 ^{abc}	0.16 ± 0.01 ^{ab}	0.02 ± 0.01 ^{bc}	0.89 ± 0.10 ^{abc}
	T	0.05 ± 0.01 ^{a}	0.06 ± 0.01 ^{cd}	0.06 ± 0.01 ^{ef}	0.12 ± 0.01 ^{de}	0.10 ± 0.01 ^{ab}	0.32 ± 0.01 ^{abc}	0.16 ± 0.01 ^{ab}	0.02 ± 0.01 ^{cde}	0.89 ± 0.09 ^{abc}
	M–I	0.05 ± 0.01 ^{a}	0.07 ± 0.01 ^{d}	0.07 ± 0.01 ^{f}	0.13 ± 0.01 ^{e}	0.11 ± 0.01 ^{ab}	0.35 ± 0.04 ^{c}	0.17 ± 0.02 ^{ab}	0.02 ± 0.01 ^{cde}	0.98 ± 0.10 ^{c}
	M–T	0.04 ± 0.01 ^{a}	0.05 ± 0.01 ^{bcd}	0.06 ± 0.01 ^{cdef}	0.11 ± 0.01 ^{cde}	0.09 ± 0.01 ^{a}	0.31 ± 0.01 ^{abc}	0.15 ± 0.01 ^{a}	0.02 ± 0.01 ^{c}	0.84 ± 0.09 ^{abc}
	I–T	0.05 ± 0.01 ^{a}	0.06 ± 0.01 ^{d}	0.07 ± 0.01 ^{f}	0.13 ± 0.01 ^{e}	0.10 ± 0.01 ^{ab}	0.34 ± 0.01 ^{bc}	0.16 ± 0.01 ^{ab}	0.03 ± 0.01 ^{ef}	0.95 ± 0.10 ^{bc}
Vacuum Drying 90 °C	M	0.06 ± 0.01 ^{a}	0.07 ± 0.01 ^{d}	0.05 ± 0.01 ^{bcdef}	0.12 ± 0.01 ^{de}	0.11 ± 0.01 ^{ab}	0.36 ± 0.01 ^{c}	0.16 ± 0.01 ^{ab}	0.02 ± 0.01 ^{cde}	0.95 ± 0.11 ^{bc}
	I	0.05 ± 0.01 ^{a}	0.05 ± 0.01 ^{abcd}	0.04 ± 0.01 ^{bcde}	0.11 ± 0.01 ^{cde}	0.10 ± 0.01 ^{ab}	0.33 ± 0.01 ^{abc}	0.15 ± 0.01 ^{a}	0.03 ± 0.01 ^{def}	0.85 ± 0.10 ^{abc}
	T	0.06 ± 0.01 ^{a}	0.05 ± 0.01 ^{abcd}	0.03 ± 0.01 ^{ab}	0.12 ± 0.01 ^{de}	0.11 ± 0.01 ^{ab}	0.37 ± 0.01 ^{c}	0.17 ± 0.01 ^{ab}	0.03 ± 0.01 ^{f}	0.95 ± 0.11 ^{bc}
	M–I	0.07 ± 0.01 ^{a}	0.05 ± 0.01 ^{abcd}	0.04 ± 0.01 ^{bcd}	0.11 ± 0.01 ^{cde}	0.10 ± 0.01 ^{ab}	0.35 ± 0.01 ^{c}	0.16 ± 0.01 ^{ab}	0.03 ± 0.01 ^{f}	0.92 ± 0.10 ^{bc}
	M–T	0.07 ± 0.01 ^{a}	0.05 ± 0.01 ^{abcd}	0.05 ± 0.01 ^{bcdef}	0.12 ± 0.01 ^{de}	0.11 ± 0.01 ^{ab}	0.36 ± 0.01 ^{c}	0.17 ± 0.01 ^{ab}	0.02 ± 0.01 ^{cd}	0.94 ± 0.11 ^{bc}
	I–T	0.07 ± 0.01 ^{a}	0.03 ± 0.01 ^{abc}	0.01 ± 0.01 ^{a}	0.09 ± 0.01 ^{abc}	0.10 ± 0.01 ^{ab}	0.38 ± 0.01 ^{c}	0.16 ± 0.01 ^{ab}	0.04 ± 0.01 ^{g}	0.88 ± 0.12 ^{abc}

M – Maltodextrin, I – Inulin, T – Trehalose, M–I – Maltodextrin – Inulin, M–T – Maltodextrin – Trehalose, I–T – Inulin-Trehalose; $^{a–g}$ the same letters within a column indicate no statistically significant differences (HSD Tukey test, $p \leq 0.05$).
